# Ethnicity‐based name partitioning for author name disambiguation using supervised machine learning

**DOI:** 10.1002/asi.24459

**Published:** 2021-02-23

**Authors:** Jinseok Kim, Jenna Kim, Jason Owen‐Smith

**Affiliations:** ^1^ Institute for Research on Innovation & Science, Survey Research Center, Institute for Social Research University of Michigan Ann Arbor Michigan USA; ^2^ School of Information Sciences University of Illinois at Urbana – Champaign Champaign Illinois USA; ^3^ Department of Sociology, Institute for Social Research University of Michigan Ann Arbor Michigan USA

## Abstract

In several author name disambiguation studies, some ethnic name groups such as East Asian names are reported to be more difficult to disambiguate than others. This implies that disambiguation approaches might be improved if ethnic name groups are distinguished before disambiguation. We explore the potential of ethnic name partitioning by comparing performance of four machine learning algorithms trained and tested on the entire data or specifically on individual name groups. Results show that ethnicity‐based name partitioning can substantially improve disambiguation performance because the individual models are better suited for their respective name group. The improvements occur across all ethnic name groups with different magnitudes. Performance gains in predicting matched name pairs outweigh losses in predicting nonmatched pairs. Feature (e.g., coauthor name) similarities of name pairs vary across ethnic name groups. Such differences may enable the development of ethnicity‐specific feature weights to improve prediction for specific ethic name categories. These findings are observed for three labeled data with a natural distribution of problem sizes as well as one in which all ethnic name groups are controlled for the same sizes of ambiguous names. This study is expected to motive scholars to group author names based on ethnicity prior to disambiguation.

## 1 INTRODUCTION

A big challenge in managing digital libraries is that author names in bibliographic data are ambiguous because many authors have the same names (homonyms) or variant names are recorded for the same authors (synonyms). One study estimates that about two‐thirds of author names in PubMed, the largest biomedicine digital library, are vulnerable to either or both of these two ambiguity types (Torvik & Smalheiser, 2009). Research findings obtained by mining bibliographic data can be distorted by merged and/or split author identities due to incorrect disambiguation (Fegley & Torvik, 2013; Kim & Diesner, 2015, 2016; Schulz, 2016). In addition, digital library users query author names most frequently (Islamaj Dogan, Murray, Névéol, & Lu, 2009). This means that the users will receive inaccurate information about research production, citation, and collaboration for authors if author name ambiguity is not properly resolved (Harzing, 2015; Strotmann & Zhao, 2012).

To address the challenge, researchers have proposed a variety of author name disambiguation (AND) methods. Some scholars have used heuristics such as string‐based matching (e.g., names that have the same full surname and forename initials are assumed to represent the same author), which is the most widely used approach in bibliometrics (Milojević, 2013). Others have developed rule‐based programming and supervised/unsupervised machine learning techniques, as systemically reviewed in several papers (Ferreira, Gonçalves, & Laender, 2012; Hussain & Asghar, 2017; Sanyal, Bhowmick, & Das, 2019; Smalheiser & Torvik, 2009). In industry, several bibliographic data providers such as DBLP, Scopus, and Web of Science have disambiguated author names to improve their service quality (Kawashima & Tomizawa, 2015; Kim, 2018; Ley, 2009; Zhao, Rollins, Bai, & Rosen, 2017), while others still rely on the name string matching to output author‐related search results.

Despite the differences in methods and datasets, a few AND studies have observed that some ethnic name groups (ENGs) (e.g., Chinese names) are more difficult to disambiguate than others (Deville et al., 2014; Kim & Diesner, 2016; Strotmann & Zhao, 2012; Torvik & Smalheiser, 2009; Wu & Ding, 2013). This implies that author names may be better disambiguated if their associated ethnicities are considered as inputs in disambiguation models. But this possibility has been little explored. First, the observations made in several studies that certain ethnic names are harder to disambiguate are based on post hoc evaluations of AND results. In other words, many of those studies did not integrate ethnic name partitions during machine learning. A very small number of studies have divided names into subgroups in their disambiguation model building (Chin et al., 2013; Louppe, Al‐Natsheh, Susik, & Maguire, 2016) and evaluation process (Lerchenmueller & Sorenson, 2016). But their ethnic name categories are limited in number (e.g., dichotomy of Chinese vs. non‐Chinese; Caucasian, Asian, and Hispanic) or mixed up with racial distinctions based on the U.S. Social Security information (e.g., White, Black, Hispanic, Asian, etc.). Such racial classifications can be inappropriate for bibliographic data in which author names come from diverse regions around the world. In addition, those studies have typically used a single labeled data source, which makes it hard to expand and generalize their findings to other AND scenarios.

This study aims to empirically evaluate the effect ethnic name partitioning has on AND. In this study, AND is a task to assign either “match” or “nonmatch” label to a pair of author name instances. For this, specifically, name instances are grouped into a block that share the same first forename initial and full surname and pairwisely compared within the block for their similarities over a set of features (e.g., coauthor name) to produce similarity scores. Machine learning algorithms combine the scores to learn weights of each feature to decide if a given pair of instances to refer to the same author or not. Although our work is motivated by the studies reviewed above and follows their common data preprocessing, blocking and machine learning steps, this paper differs from them in three important ways. First, this study evaluates AND performance by four different machine learning algorithms applied to four different labeled datasets before and after inclusion of a standard ENG partition. Here, a name instance is assigned to an ENG based on a name ethnicity classification system, *Ethnea*. Second, unlike traditional labeled data in which a specific ENG (i.e., Chinese) dominates, this study disambiguates new labeled data in which all ENGs are controlled to have the same numbers of instances, to demonstrate that performance changes induced by ethnic name partitioning may not be solely due to the well‐known relationship between the number of cases and their ambiguity (more names, more ambiguity). Third, this study shows that different combinations of features (e.g., coauthor name and title words) appear to be related to AND performance for different ENGs suggesting future directions to further improve AND performance with ambiguous ethnic names. The findings of this study can provide practical insights to researchers and practitioners who handle authority control in digital libraries. In the following sections, details on labeled data and setups for machine learning are described.

## 2 METHOD

### 2.1 Labeled data and preprocessing

To measure the effect of ethnic name partition on machine learning for AND, this study disambiguates names in four labeled datasets—KISTI, AMiner, GESIS, and UM‐IRIS. The first three datasets have been used in many AND studies to train and test machine learning algorithms (Cota, Ferreira, Nascimento, Gonçalves, & Laender, 2010; Ferreira, Veloso, Gonçalves, & Laender, 2014; Hussain & Asghar, 2018; Kim & Kim, 2018, 2020; Momeni & Mayr, 2016; Santana, Gonçalves, Laender, & Ferreira, 2017; Shin, Kim, Choi, & Kim, 2014; Wu, Li, Pei, & He, 2014; Zhu et al., 2018). The last one is added to investigate how the ethnic name partition affects AND under the condition in which all ENGs are constrained to have the same numbers of ambiguous name instances.^1^

#### 2.1.1 KISTI

Scientists at the Korea Institute of Science & Technology Information (KISTI) and Kyungsung University in Korea constructed this labeled dataset. It is made up of 41,673 author name instances that belong to 6,921 unique authors (Kang, Kim, Lee, Jung, & You, 2011).^2^

#### 2.1.2 AMiner

Researchers in China and U.S. collaborated to create this labeled data to build and evaluate AND models for a computer science digital library, AMiner (Tang et al., 2008; Wang, Tang, Cheng, & Yu, 2011).^3^ It consists of 7,528 author name instances that refer to 1,546 unique authors.

#### 2.1.3 GESIS

Scholars at the Leibniz Institute for the Social Sciences (GESIS) in Germany produced this labeled data. It contains author name instances of 5,408 unique authors (Momeni & Mayr, 2016).^4^ This study reuses the “Evaluation Set” (29,965 author name instances of 2,580 unique authors) but with a few enhancements (Kim & Kim, 2020). Each author name instance is converted into the “surname, forename” format and, through linking GESIS to its base DBLP data, is associated with the title of the paper in which it appears and the name of the conference or journal where the paper is published.

#### 2.1.4 UM‐IRIS

This dataset was generated by the researchers at the University of Michigan Institute for Research on Innovation & Science (UM‐IRIS) through matching selected name instances in publication records to an authority database, ORCID (Kim & Owen‐Smith, 2021). First, author full names (e.g., “Brown, Michael”) that appear 50 times or more in MEDLINE‐indexed publications published between 2000 and 2019 were listed.^5^ Then, all instances of each selected full name (e.g., 158 instances of “Brown, Michael” in MEDLINE) and their associated publication metadata were compared to 6 million researcher profiles in ORCID.^6^ If an instance had a single match in the publication list of an ORCID researcher profile (matching on full name, paper title, and publication venue), the matched researcher's ORCID id was assigned as an author label to the instance. Next, among the ORCID id‐linked instances, those whose full names are associated with five or more ORCID ids (e.g., six unique ORCID researchers share the name “Brown, Michael” which appear 158 times in MEDLINE) were randomly selected to produce 1,000 name instances for each of six ENGs. The resulting data contain 6,000 instances of 822 authors.

Four features—author name, coauthor name(s), paper title, and publication venue—are used as machine learning features because they have been widely used in algorithmic AND studies (Schulz, 2016; Song, Kim, & Kim, 2015) and are commonly available in the four labeled datasets. The string of each feature is stripped of nonalphabetical characters, converted into ASCII format, and lowercased. For title words, common English words like “the” and “to” are removed (i.e., stop‐word listed) using the dictionary in Stanford NLP^7^ and stemmed (e.g., “solution” → “solut”) using the Porter's algorithm.^8^ Name instances in KISTI are converted into the full surname and first forename initial format (“Wang, Wei” → “Wang, W”) to make them more ambiguous (see Kim & Kim, 2020).

### 2.2 ENG tagging

This study assigns an ENG tag to a name instance in each labeled dataset using the author name ethnicity classification database, Ethnea, developed by Torvik and Agarwal (2016).^9^ Ethnea is a collection of more than 9 million author name instances that are tagged one of 26 ENG classes based on the name's association with national‐level geo‐locations.^10^ For example, “Wang, Wei” is classified as “Chinese” as it is most frequently associated with organizations in China. However, Ethnea makes no distinctions based on any anthropological, cultural, or linguistic characteristics of authors. Instead it relies entirely on observations of names and geo‐locations of their frequently associated institutions so an author named “Wang, Wei” who was born in the United States and has never visited China would still be assigned a “Chinese” tag. We link all four labeled datasets to Ethnea and, if a matched name is found, assign that name's ENG tag to all its observed instances. If a queried name does not have a match in Ethnea, we search again using only the surname and assign the modal Ethnea ENG tag associated with it to all its instances. Table 1 summarizes the frequencies and ratios of ENG tags assigned by Ethnea to author name instances in each labeled dataset.

**TABLE 1:** Summary of ethnic name group (ENG) frequencies in labeled data

Labeled data (no. of instances)							
KISTI (41,605)		AMiner (7,528)		GESIS (29,965)		UM‐IRIS (6,000)	
ENG	Ratio (%)	ENG	Ratio (%)	ENG	Ratio (%)	ENG	Ratio (%)
Chinese	50.1	Chinese	64.2	Chinese	56.2	Chinese	16.67
English	15.6	English	17.0	German	14.8	English	16.67
Indian	9.5	Indian	6.3	English	7.0	German	16.67
Korean	8.0	German	5.6	Indian	4.3	Hispanic	16.67
German	3.3	Hispanic	3.1	Hispanic	3.6	Indian	16.67
Israeli	2.2	Sum	96.2	Korean	3.5	Korean	16.67
Italian	2.0	Excluded ENGs (3.8%): Japanese, Nordic, Korean, Arab		Japanese	2.8	Sum	100.00
Hispanic	1.7			Italian	1.7	Excluded ENGs (0%): None	
Japanese	1.1			Arab	1.6		
Dutch	1.0			French	1.3		
Arab	0.9			Sum	96.8		
French	0.9			Excluded ENGs (3.2%): Nordic, Hungarian, Dutch, Vietnamese, Slav, Romanian, Greek, Israeli, Turkish, Indonesian			
Sum	96.3						
Excluded ENGs (3.7%): Nordic, Slav, Greek, Romanian, Vietnamese, Null, African, Turkish, Hungarian							

Table 1 shows the list of ENGs in each labeled data. Small‐sized ENGs are excluded from analysis because most name instances in those ENGs tend to belong to a single author while a few instances referring to other author(s). When randomly split into training and test subsets for machine learning, these instances do not produce negative pairs at all.

Chinese names represent the majority of ENGs in three labeled data. This is because these labeled data were created from computer science papers where Chinese researchers are particularly large contributors. In addition, as the three datasets were designed to collate challenging names to disambiguate, Chinese names that tend to be more ambiguous than other ENGs were oversampled (Müller, Reitz, & Roy, 2017). In contrast, 6,000 instances in UM‐IRIS are evenly distributed over six ENGs. For validation and reuse, these labeled data with ENG tags are publicly available.^11^ Note that the original KISTI contains 41,673 name instances, whereas the ENG‐tagged KISTI has 41,605 instances. Such discrepancy occurs because this paper uses the revised version of KISTI that corrects record errors and duplicates in the original data (Kim, 2018).

### 2.3 Machine learning process

Machine learning methods for AND can be divided into two groups: author assignment and author grouping (Ferreira et al., 2012). While the former aims to assign an author name instance to one of pre‐disambiguated author name clusters, the latter aims to group all and only instances that belong to the same authors. This study takes the latter approach in evaluating the effect of ENG on AND. Specifically, author name instances in each labeled dataset are pairwise compared to assess whether a given instance pair plausibly represents the same author (a match) or not (a nonmatch). Although some scholars take a further step to cluster pairwise comparisons (e.g., Kim & Kim, 2018; Levin, Krawczyk, Bethard, & Jurafsky, 2012; Louppe et al., 2016; Santana et al., 2017), this study only evaluates disambiguation performance at a pair level (i.e., classification), following the practice of previous AND studies (e.g., Han, Giles, Zha, Li, & Tsioutsiouliklis, 2004; Song et al., 2015; Treeratpituk & Giles, 2009; Vishnyakova, Rodriguez‐Esteban, Ozol, & Rinaldi, 2016).

As the first machine learning step, author name instances in each labeled dataset are randomly divided into training (50%) and test (50%) subsets. Then, instances in each subset are put into blocks in which all member instances share the same full surname and first forename initial (e.g., “Wang, W”). Only instances in the same block are compared for disambiguation. This blocking is typical in AND studies because it reduces computational complexity with only slight performance degradation (Kim, Sefid, & Giles, 2017; Torvik & Smalheiser, 2009). Next, instance pairs in the same block are compared to establish their similarity over four other data features: author name, coauthor name(s), paper title, and publication venue. To quantify how much a pair is similar over a feature, this study calculates the cosine similarity of term (*n*‐gram) frequency for each feature (Han, Zha, & Giles, 2005; Kim & Kim, 2020; Levin et al., 2012; Louppe et al., 2016; Santana, Gonçalves, Laender, & Ferreira, 2015; Treeratpituk & Giles, 2009). Specifically, the string of a feature is converted into an array of 2–4‐grams (e.g., author name “Wang, Wei” → “wa|an|ng|gw|we|ei|wan|ang|ngw|gwe|wei|wang|angw|ngwe|gwei”). After the conversion, two *n*‐gram arrays of an instance pair are compared to produce a cosine similarity score for the feature.

Besides the four basic features, ENGs are used as a feature set for ENG‐aware disambiguation. For this, especially, an instance pair's ENG is encoded into a binary value (i.e., one‐hot encoding) for a predefined set of ENGs.^12^ For example, in AMiner, a pair of name instances (“Wang, Wei” and “Wang, W.”) is assigned either “Yes” or “No” for each of five ethnicities—Chinese (“Yes”), English (“No”), Indian (“No”), German (“No”), and Hispanic (“No”)—as shown in Table 1. Table 2 shows examples of the cosine similarity scores calculated over four features and ENG encoding results for instance pairs.

**TABLE 2:** A mock‐up example of cosine similarity scores for instance pairs over four features and ethnic name group (ENG) encoding

Pairs	Feature							Label
	Author name	Coauthor name	Paper title	Pub. venue	ENG 1	ENG 2	ENG 3	
Pair 1	0.97	0.89	0.67	0.12	Yes	No	No	Match
Pair 2	1.00	0.24	0.46	0.00	No	Yes	No	Nonmatch
Pair 3	0.65	0.07	0.00	0.80	No	No	Yes	Nonmatch
Pair 4	0.58	0.08	0.00	0.00	Yes	No	No	Nonmatch

We focus on four algorithms—gradient boosting (GB), logistic regression (LR), naïve Bayes (NB), and random forest (RF)—for supervised machine learning that have been widely used as baselines or best performing methods in AND studies (e.g., Han et al., 2004; Kim & Kim, 2020; Kim, Sefid, Weinberg, & Giles, 2018; Louppe et al., 2016; Song et al., 2015; Torvik & Smalheiser, 2009; Treeratpituk & Giles, 2009; Vishnyakova et al., 2016; Wang et al., 2012). In the first scenario, they are trained on the list of similarity scores and labels, as shown in Table 2 to learn relative weights for features and an absolute weight or threshold for instance pairs to be disambiguated without considering ENGs (→ ENG‐ignorant learning). In the second scenario, the same algorithms are trained on the list of similarity scores, ENGs, and labels (→ ENG‐aware disambiguation). Here, the ethnic name partition adds more features (dimensions) to each instance pair's feature set, allowing algorithms to combine the similarities of the expanded features. The machine learning procedure is implemented using the python Scikit‐learn package. For GB, 500 estimators are used with max depth = 9 and learning rate = 0.125. For LR, L2 regularization with class weight = 1 is used. Gaussian NB with maximum likelihood estimator is used for NB. For RF, 500 trees are used after a grid search.

Trained algorithmic models are applied to the instance pairs in test subsets in which the cosine similarity is calculated for the four basic features and, in the second scenario, ethnicities are encoded in the same fashion but explicitly include ENG information. As in Table 2, an algorithmic model receives a set of feature similarity scores and, if ENG‐aware disambiguation is conducted, a list of encoded ENGs for an instance pair to output a binary classification decision (match or nonmatch). Once trained, each algorithm produces a single score that predicts the probability of an instance pair being negative (nonmatch). If the predicted probability is above a certain threshold (>0.5), the pair is decided to be a nonmatch, whereas if below the threshold, a match.

### 2.4 Performance evaluation

We evaluate each algorithm's classification results on reserved test subsets of each labeled dataset by calculating precision and recall for positive (P; match) and negative (N; nonmatch) pairs, respectively. In addition, we calculate the F1 score as a harmonic mean of precision and recall.

Specifically, precision for positive pairs (PrecPos) measures how many predicted match pairs are correct ones (true positives [TP]) over the total number of predicted match pairs that may contain correct match pairs (TP) and incorrect match pairs (false positives [FP]). In contrast, recall for positive pairs (RecPos) measures the ratio of correct match pairs (TP) over the total number of true match pairs that may be predicted correctly as match pairs (TP) or incorrectly as nonmatch pairs (false negatives [FN]),

(1) $$\text{Precision positive}\;\left(\text{PrecPos}\right)=\frac{\text{Number of correctly predicted match}}{\text{Number of predicted match}}=\frac{\mathrm{TP}}{\left(\mathrm{TP}+\mathrm{FP}\right)},$$

(2) $$\text{Recall positive}\;\left(\text{RecPos}\right)=\frac{\text{Number of correctly predicted match}}{\text{Number of true match}}=\frac{\mathrm{TP}}{\left(\mathrm{TP}+\mathrm{FN}\right)},$$

(3) $$F1\;\text{positive}=\frac{2\times \text{PrecPos}\times \text{RecPos}}{\text{PrecPos}+\text{RecPos}}.$$

Likewise, precision for negative pairs (PrecNeg) measures how many predicted nonmatch pairs are correct ones (true negatives [TN]) over the total number of predicted nonmatch pairs that may contain correct nonmatch pairs (TN) and incorrect nonmatch pairs (FN). In contrast, recall for negative pairs (RecNeg) measures the ratio of correct nonmatch pairs (TN) over the total number of true nonmatch pairs that may be predicted correctly as nonmatch pairs (TN) or incorrectly as match pairs (FP),

(4) $$\text{Precision}\;\text{negative}\;\left(\text{PrecNeg}\right)=\frac{\text{Number of correctly predicted nonmatch}}{\text{Number of predicted nonmatch}}=\frac{\mathrm{TN}}{\left(\mathrm{TN}+\mathrm{FN}\right)},$$

(5) $$\text{Recall}\;\text{negative}\;\left(\text{RecNeg}\right)=\frac{\text{Number of correctly predicted nonmatch}}{\text{Number of true nonmatch}}=\frac{\mathrm{TN}}{\left(\mathrm{TN}+\mathrm{FP}\right)},$$

(6) $$F1\;\text{negative}=\frac{2\times \text{PrecNeg}\times \text{RecNeg}}{\text{PrecNeg}+\text{RecNeg}}.$$

The metrics are calculated on the entire set of test results for each labeled dataset. We separately calculate performance measures for different ENGs rather than averaging them across multiple ethnicity groups.

## 3 RESULTS

### 3.1 Cross‐data performance evaluation

**FIGURE 1:**
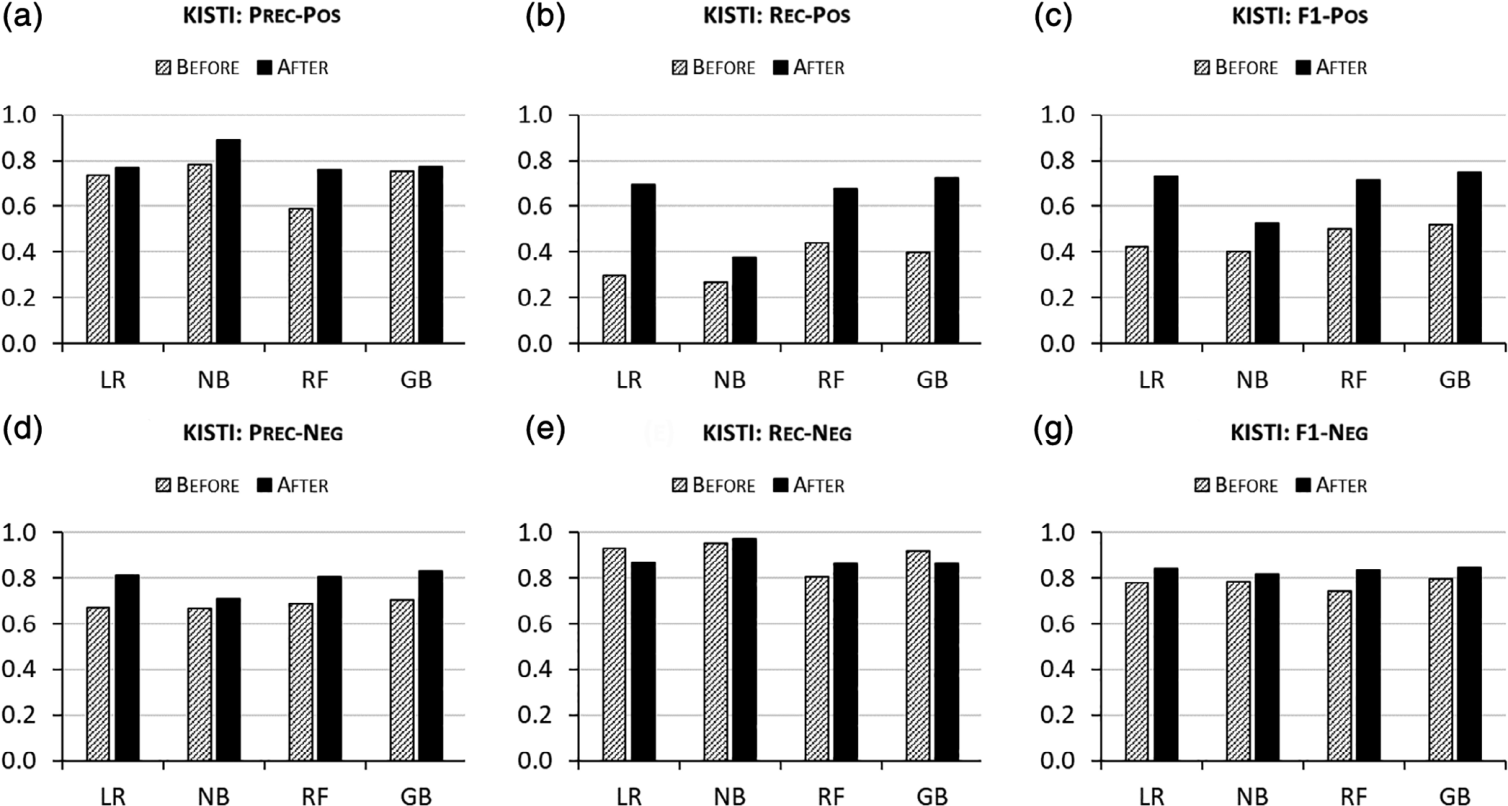
Disambiguation performances “Before” versus “After” ethnic name group (ENG)‐aware disambiguation on KISTI

Figure 1 shows disambiguation results on KISTI, reporting precision and recall *before* and *after* ENG‐aware disambiguation by four algorithms—GB, LR, NB, and RF. Figure 1a shows that when ENGs are included as features, the algorithms tend to produce better precision in the prediction of positive (match) pairs than when they are not considered. This is shown by black bars (“After”) being higher than stripped bars (“Before”) in Figure 1a. This observation indicates that ethnic name partitioning helps algorithms increase the ratio of TP among predicted positive pairs (= TP + FP). This can be confirmed by checking the numbers of TP and FP pairs in Table 3. For example, when trained only on the four basic (non‐ENG) features, LR predicts that 76,201 (= TP + FP = 55,998 + 20,203) pairs refer to the same authors (match) and 73.49% of the predictions are right [= TP/(TP + FP)]. After trained on the same but ENG‐tagged data, however, it predicts 170,432 pairs to be match sets, increasing its prediction accuracy this time to 77.08%.

**TABLE 3:** Numbers of correctly or incorrectly predicted pairs for positive and negative pairs by four algorithms on KISTI test data

Algorithm	ENGs considered	No. of pairs	TP	FN	FP	TN
LR	Before	483,029 (P: 189,375) (N: 293,654)	55,998	133,377	20,203	273,451
	After		131,364	58,011	39,068	254,586
NB	Before		50,782	138,593	14,267	279,387
	After		71,020	118,355	8,775	284,879
RF	Before		82,727	106,648	57,532	236,122
	After		128,105	61,270	40,479	253,175
GB	Before		75,195	114,180	24,725	268,929
	After		136,859	52,516	39,888	253,766

Abbreviations: FN, false negatives; FP, false positives; GB, gradient boosting; LR, logistic regression; N, negative; NB, naïve Bayes; P, positive; RF, random forest; TN, true negatives; TP, true positives.

Ethnic name partitioning also reduces the number of falsely predicted nonmatch cases (FN), increasing recall in Figure 1b. Performance gains by ENG‐aware disambiguation are more pronounced for recall than for precision, as evidenced by larger differences between “Before” and “After” bars for recall (Figure 1b) than those for precision (Figure 1a). In other words, ENG‐aware disambiguation across four common algorithms appears to reduce FN predictions more than TP predictions, potentially providing better performance for applications (such as network analysis) that are particularly sensitive to biases due to erroneous “lumping” of name instances that actually refer to different individuals. The improvements in precision and recall together increase the F1 scores by ENG‐aware disambiguation (Figure 1c).

ENG‐aware disambiguation also does a better job of accurately predicting nonmatch (negative pair) cases. The “After” bars are taller than those of “Before” in Figure 1d. Unlike the positive pair prediction in which ENG‐aware disambiguation works in favor of both precision and recall by all algorithms, however, the performance gains in precision for negative pairs come with slightly decreased recall by GB and LR in Figure 1e. This means that while disambiguation models by GB and LR trained on ENG‐added features are good at increasing the numbers of true nonmatch pairs among predicted nonmatch pairs (= TN + FN), they incorrectly predict that true nonmatch pairs match (FP predictions) more frequently than when they are trained on the four basic features alone. Reduced recall for negative pair prediction is, however, offset by increased precision, leading to the F1 scores by ENG‐aware disambiguation being better than those by ENG‐blind one in Figure 1f. Meanwhile, NB and RF still obtain improvements in both precision and recall as well as F1.

**FIGURE 2:**
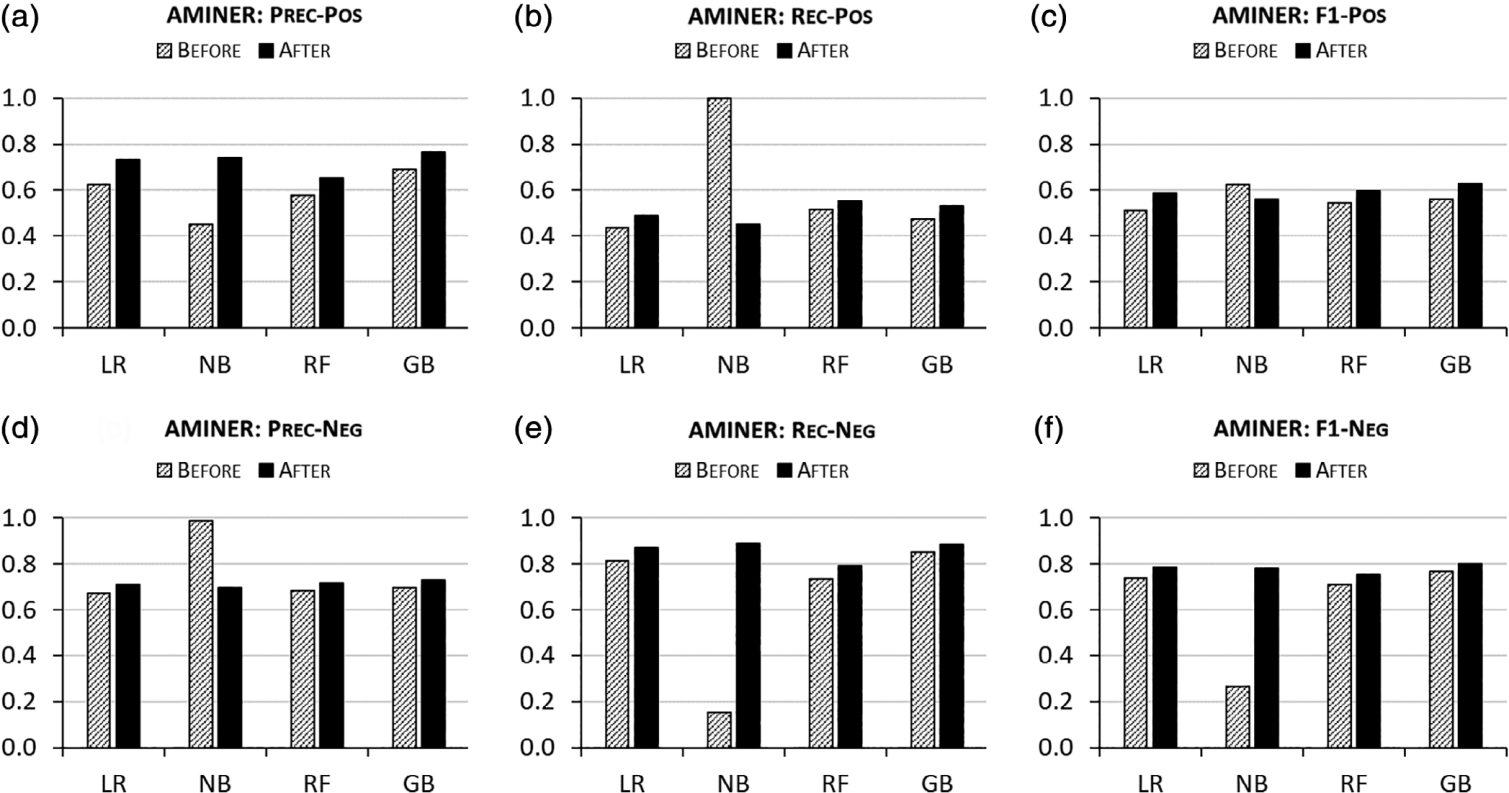
Disambiguation performances “Before” versus “After” ethnic name group (ENG)‐aware disambiguation on AMiner

**FIGURE 3:**
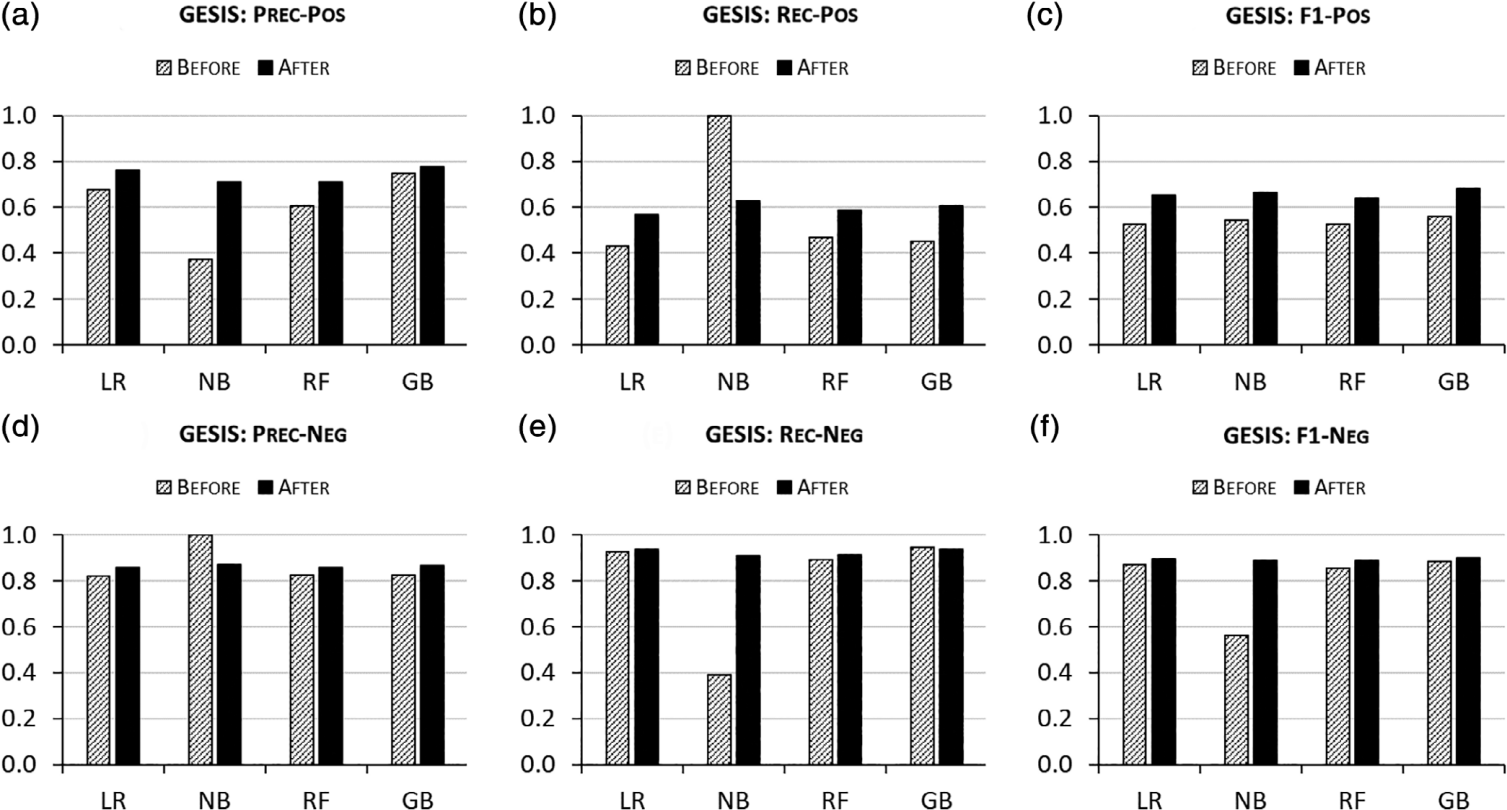
Disambiguation performances “Before” versus “After” ethnic name group (ENG)‐aware disambiguation on GESIS

**FIGURE 4:**
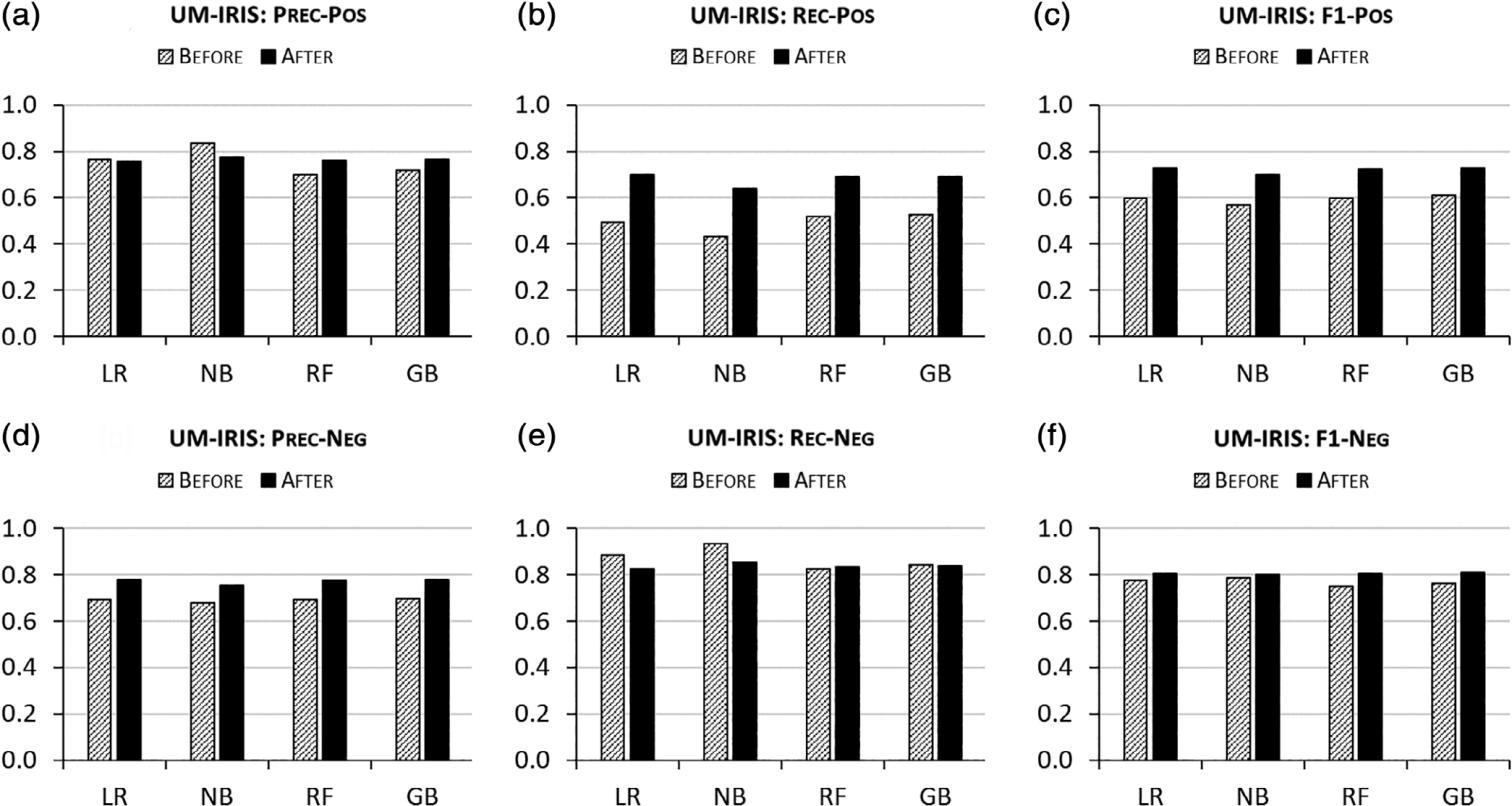
Disambiguation performances “Before” versus “After” ethnic name group (ENG)‐aware disambiguation on UM‐IRIS

Algorithmic performances are also enhanced by ENG‐aware disambiguation on AMiner, GESIS, and UM‐IRIS. Figures 2, 3, 4 report that the algorithms trained on ENG‐tagged data perform better than those trained only on the basic features across almost all metrics for both positive and negative pairs. NB models prove the exception, producing worse results in recall for positive pairs and in precision for negative pairs after ethnic name partitioning. However, this degraded performance is offset by increased precision for positive pairs and increased recall for negative pairs, respectively, so the overall performance metric (F1), which equally weights precision and recall, indicates an overall improvement due to the inclusion of ENG features.

### 3.2 Performance evaluation per ENG

ENG‐aware disambiguation produces substantial improvements in both precision and recall for predicting match and nonmatch instance pairs in different labeled datasets. But are those improvements uniform across different ENGs? If not, a more nuanced approach to model evaluation may be necessary. To answer this question, we compare performance changes due to ENG‐aware disambiguation within ENG groups. For this, precision, recall, and F1 scores for positive and negative pairs predicted by four algorithms are calculated separately for instance pairs that belong to the same ENG in each of four labeled data: 4 algorithms × 4 data = 16 evaluations. Presenting all the results at the same time would consume too much space in this paper. So, we present RF predictions on the GESIS dataset as an illustration for the purposes of this discussion. Reports of other algorithms and data are presented in Supporting Information attached to this paper.

**FIGURE 5:**
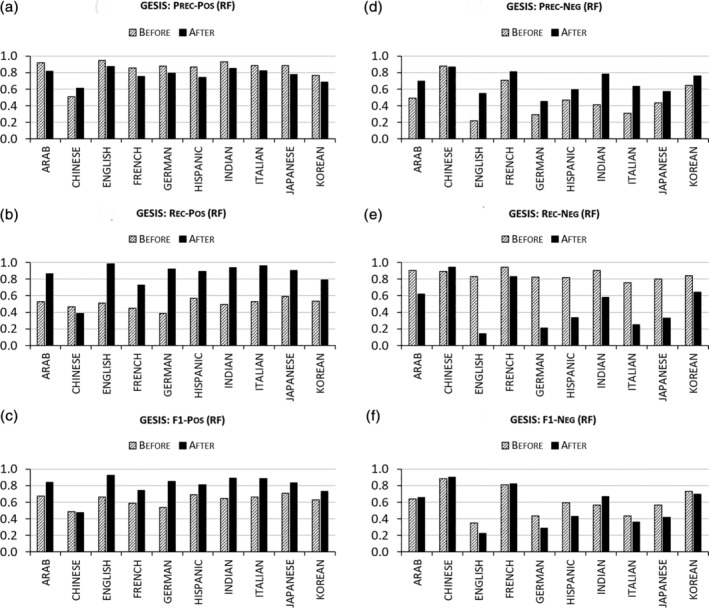
Disambiguation performances per ethnic name group (ENG) “Before” versus “After” ENG‐aware disambiguation by random forest on GESIS

Figure 5 shows the by ENG performance metrics for the RF algorithm trained on GESIS with and without ENG‐aware disambiguation. The ENG‐aware disambiguation leads to better precision (positive pairs; Figure 5a) and recall (negative pairs; Figure 5e) for Chinese names but worse precision (positive pairs) and recall (negative pairs) for other ethnicities. In contrast, name disambiguation for Chinese names results in lower recall (positive pairs; Figure 4b) and precision (negative pairs; Figure 4d) than those for other ENGs. Similar patterns are observed for other algorithms tested on GESIS (see Figures S5–S8, Supporting Information). This suggests that the effect of ENG‐aware disambiguation occurs in different ways for different ENGs. Thus, its application can be beneficial in some instances but detrimental in others. Variations in the effects of ENG‐aware disambiguation on precision and recall for positive and negative pair prediction across ethnicity groups suggest that care must be taken to design disambiguation strategies that fit particular analytic or empirical needs.

**FIGURE 6:**
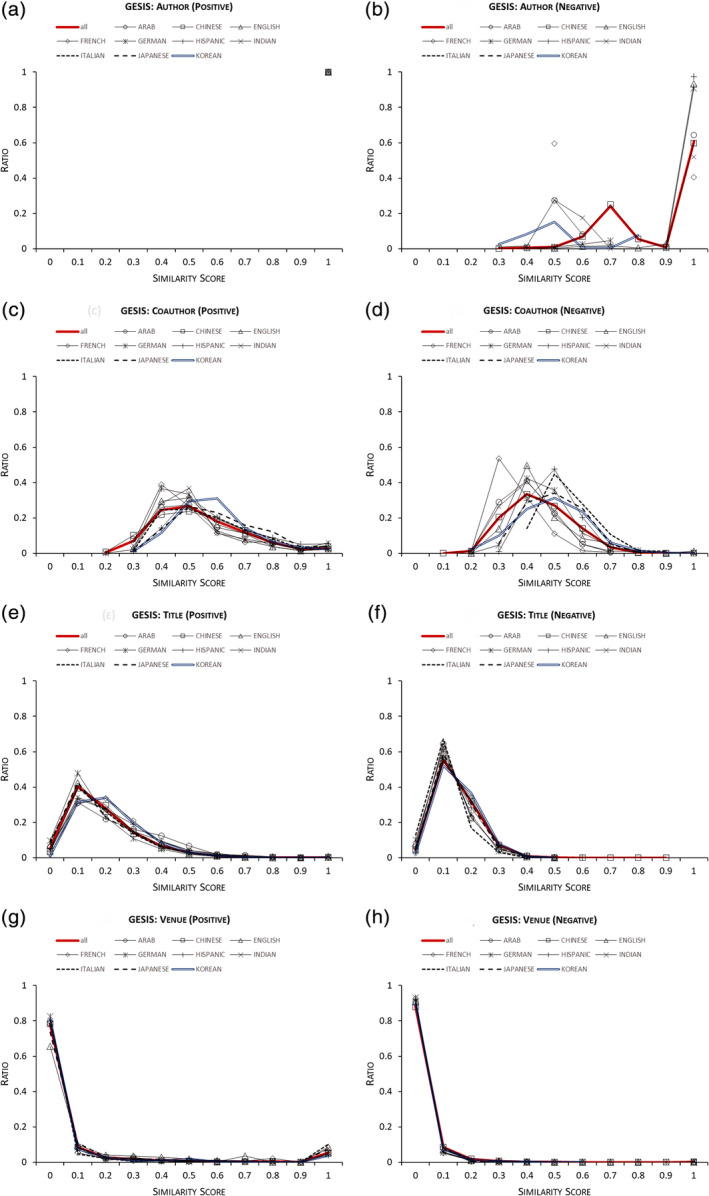
Feature similarity score distributions per ethnic name group (ENG) for positive and negative pairs in GESIS test data [Color figure can be viewed at wileyonlinelibrary.com]

These observations can be explained as follows. ENGs have different distributions of similarity scores over the four basic (nonethnicity) features we use. Figure 6 presents the feature similarity score distributions per ENG for positive (left) and negative (right) pairs in the GESIS test data. Training and test subsets show similar distributions in each labeled dataset. For visual simplicity, a score is rounded up into nearest bins with intervals of 0.1 on *x*‐axis and the ratios of the numbers of scores in the same bin over all scores are plotted on *y*‐axis. A solid red line represents the distribution of all instance pairs regardless of ENG.

In Figure 6, each ENG has different distributions of, for example, “coauthor” similarity scores for both positive and negative pairs (Figure 6c,d). So, the four algorithms come to use different “coauthor” similarity score distributions in ENG‐aware disambiguation. Such heterogeneous distributions also occur for other features but with different variations of differences. For example, “Venue” distributions in Figure 6g,h differ less across ENGs than do “Coauthor” distributions. Because ENG‐aware disambiguation allows training and testing on different feature similarity score distributions for each ENG, the algorithms combine features using different weightings for each ethnicity, producing different predictions for name pairs with the same feature similarity scores but different ENG tags. In other words, this method takes into account the likelihood that researchers in different ENGs organize their scientific work differently, favoring distinct coauthorship and publication venue patterns. This also occurs in disambiguation of other labeled data, whose feature similarity score distributions are reported in Figures S17–S20.

Figure 5 also shows that some ENGs manifest substantial improvements in recall for positive pairs (Figure 5b) but degraded recall for negative pairs (Figure 5e). This might be explained in two ways. In our “Before” (ENG‐unaware) case, algorithms combine features to produce per‐feature weights for positive pairs based on feature similarity scores aggregated across multiple ENGs that can have very different feature distributions. Such aggregated distributions cannot effectively capture the single match patterns specific to each ENG, which seem to lead models to falsely predict positive pairs as negative ones (FN), reducing the recall for positive pairs. Conversely, increased recall for positive pairs after ENG‐aware disambiguation means that the algorithms trained and tested on ENG‐tagged data successfully produce per‐feature weights optimized to each ENG, thus making better predictions that push up the recall scores for many ENGs.

Second, decreased negative pair recall after ENG‐aware disambiguation means that the algorithms trained and tested on ENG‐tagged data fail to produce proper per‐feature weights for accurately predicting nonmatch for known negative pairs. When the algorithms are trained only on the four basic (nonethnicity) features, they do a better job of predicting nonmatch pairs based on aggregated feature similarity distributions that are invariant across particular ENGs. In other words, feature distributions aggregated across ENGs appear to be more effective for predicting negative case pairs while ENG‐aware disambiguation techniques more accurately capture positive pairs.

These observations imply that disambiguation models for positive pair prediction would be improved by ENG‐aware procedures, while nonmatch patterns for negative pair prediction can aggregate across ENGs (Kim & Kim, 2018). Table 4 shows that in the GESIS training data, each ENG has different sizes of positive and negative (pairwise) pairs. Chinese name instances produce the largest numbers of positive (≈146 K) and negative pairs (≈551 K), while Italian name instances generate around a few thousand positive and a few hundred negative pairs. In other training data, Chinese pairs constitute substantially large proportions (KISTI: 71.28% and AMiner: 91.26%) or over one‐third (UM‐IRIS: 37.54%) of all negative pairs, while other ENG pairs make up small or less‐than‐expected (approximately 17% per ENG in UM‐IRIS) proportions. In contrast, the numbers of positive pairs are less concentrated (GESIS, KISTI, and AMiner) or more evenly distributed (UM‐IRIS) for positive pairs than those for negative pairs.

**TABLE 4:** Distributions of positive and negative name instance pairs per ethnic name group (ENG) in GESIS training data

ENG	Positive pairs	Ratios	Negative pairs	Ratios
Arab	1,559	0.66	1,203	0.21
Chinese	145,969	62.22	551,150	93.96
English	16,020	6.83	2,147	0.37
French	1,948	0.83	3,118	0.53
German	37,373	15.93	13,092	2.23
Hispanic	5,887	2.51	1,907	0.33
Indian	6,708	2.86	2,654	0.45
Italian	3,489	1.49	686	0.12
Japanese	8,853	3.77	2,608	0.44
Korean	6,792	2.90	7,994	1.36
Total	234,598	100	586,559	100

**TABLE 5:** Distributions of positive and negative name instance pairs per ethnic name group (ENG) in KISTI training data

ENG	Positive pairs	Ratios	Negative pairs	Ratios
Arab	6,015	3.21	37	0.01
Chinese	53,519	28.55	209,807	71.28
Dutch	5,448	2.91	84	0.03
English	45,054	24.04	25,137	8.54
French	2,686	1.43	625	0.21
German	11,065	5.90	4,114	1.40
Hispanic	3,729	1.99	1,680	0.57
Indian	33,742	18.00	19,043	6.47
Israeli	9,699	5.17	460	0.16
Italian	9,598	5.12	283	0.10
Japanese	1,588	0.85	580	0.20
Korean	5,284	2.82	32,474	11.03
Total	187,427	100	294,324	100

As noted above for Figure 5 and observed in other labeled data (see Figures S1–S16), the algorithms work better in finding more TN pairs even for non‐Chinese name pairs when they are trained on data in which ethnic name partitioning is not performed (“Before”) and, thus, negative pairs are dominated by Chinese ones as shown in Tables 4, 5, 6. This implies that the nonmatch patterns in Chinese name pairs are applicable to predicting nonmatch pairs for other ENGs. In contrast, during ENG‐aware disambiguation, the algorithms come to rely on the small‐size negative pairs that may skew or distort true nonmatch patterns for some ENGs. This seems to result in the decreased recall in predicting negative pairs (i.e., many TNs classified as FPs, which reduces precision for positive pair prediction), while increasing slightly precision in predicting negative pairs.

**TABLE 6:** Distributions of positive and negative name instance pairs per ethnic name group (ENG) in AMiner training data

ENG	Positive pairs	Ratio (%)	Negative pairs	Ratio (%)
Chinese	38,958	61.88	76,781	91.26
English	13,758	21.85	2,414	2.87
German	2,603	4.13	748	0.89
Hispanic	1,933	3.07	380	0.45
Indian	5,701	9.06	3,815	4.53
Total	62,953	100	84,138	100

Despite the aforementioned conflicting changes in precision and recall per ENG, the overall performance by the four algorithms on the whole test set are shown in Figures 1, 2, 3, 4 to substantially increase across the four labeled data after ethnic name partitioning is included in machine learning. One reason would be that performance gains outweigh losses at each ENG level overall. Another reason would be that especially for KISTI, AMiner, and GESIS, the improved performances in disambiguating Chinese that constitute the majority of name instances may affect the overall evaluation results. As shown by the case of UM‐IRIS in which ENG sizes are controlled to be equal, however, the overall performance improvements can be observed for all the ENGs by ENG‐aware disambiguation. As such, this study illustrates that the ethnic name partition can be truly effective in improving disambiguation performances.

## 4 DISCUSSION

These results suggest that AND tasks may produce better results by using ethnic name partition in machine learning. Considering that adding more features can improve generally machine learning performances, the enhanced disambiguation performances by ENG partitioning might not be a surprise. With that said, the real contribution of this study would be that it demonstrates many machine learning based disambiguation models have a potential to be improved by introducing ethnic name grouping into ambiguous data without additional collection of feature information.

To fully realize this potential, however, a few issues need to be addressed. First, ENG tagging can be a non‐trivial task that requires an intricate algorithmic technique itself. Thanks to the ENG classification system developed and publicly shared by Torvik and Agarwal (2016), this study could assign ENGs to the names in four labeled data. Although Ethnea was constructed based on more than 9 million author name instances in PubMed, the world largest biomedicine library, it is unknown how well it can help us tag ENGs to names in other fields. Ideally, Ethnea may be updated regularly to reflect new author names entering bibliographic data in various fields. Practically, further research may be focused on finding out a set of ENGs that are most influential in improving disambiguation results and thus simplifying ENG tagging for AND (e.g., Chinese vs. non‐Chinese).

Second, the findings of this study were based on three labeled data (KISTI, AMiner, and GESIS) in which Chinese names are dominant and the overall performance improvements were heavily affected by those for Chinese name instances. To overcome such an imbalance of instance distribution in labeled data, a new labeled data (UM‐IRIS) were created in a way that six ENGs have the same amount of ambiguous name instances. Disambiguation results from the new labeled data were in line with those from other three labeled data. In addition, all ENGs including Chinese were able to obtain gains in disambiguation performances. But all these findings were obtained from small‐sized labeled data, whether they are biased or controlled for ENG sizes. So, it is still unknown whether such improvements are achievable in AND for large‐scale bibliographic data in which ENG composition may be quite different from those in the labeled data used in this paper.

Another issue would be that there can be other features than the four used in this study that can lead ethnic name partition to different AND performances. For example, English authors may appear in publication records that are more complete in affiliation information and use more diverse title terms. Meanwhile, Chinese authors may tend to work with coauthors who have similar names in same institutions. Various features need to be explored to study further the impact of ethnic name partition on AND.

Fourth, ENG‐aware disambiguation may be beneficial for positive pair prediction but not so much for negative pair prediction. This was illustrated in Figures 5 and S1–S16 by the dramatically decreased recall in negative pair predictions for many ENGs. It was contrasted with the substantial increase of precision in positive pair prediction for those ENGs. This study speculates that by ethnic name partitioning, classifiers become stricter for Chinese pairs while relaxed for other ENG pairs. In other words, a pairs of Chinese instances that would be classified as “match” before partitioning are classified as “nonmatch” after partitioning (precpos↑, recpos↓), while “nonmatch” pairs of other ENG instances as matched ones (precpos↓, recpos↑). This might be because while some Chinese pairs sharing coauthor names, venue names, or title words refer to different authors, other ethnic names sharing the features are more likely to represent the same authors (see Figure 6b,d,f,h in which Chinese name pair share is denoted in square). During training, such different similarity patterns are mixed up before partitioning but distinguished after it. Another conjecture is that due to the relaxed classification after partitioning, TN pairs of other‐than‐Chinese ENGs are falsely classified as FP pairs (precpos↓, recneg↓). As the sizes of negative pairs in most non‐Chinese ENGs are smaller than positive pairs (see Tables 4, 5, 6, 7), misclassified negative pairs have larger impacts on recneg than on precpos across the ENGs. But these conjectures are based on the observations on labeled data in which Chinese name instances are prevalent. Using an additional labeled data with controlled ENG sizes, however, the conjecture has been confirmed. But only six ENGs in a small dataset were considered for analysis. More ENGs need to be investigated to check if this conjecture holds good under the different combinations of ENGs.

**TABLE 7:** Distributions of positive and negative name instance pairs per ethnic name group (ENG) in UM‐IRIS training data

ENG	Positive pairs	Ratio (%)	Negative pairs	Ratio (%)
Chinese	1,517	10.60	7,261	37.54
English	2,185	15.26	459	2.37
German	3,673	25.66	1,917	9.91
Hispanic	2,416	16.88	970	5.01
Indian	2,080	14.53	3,803	19.66
Korean	2,445	17.08	4,933	25.50
Total	14,316	100	19,343	100

## 5 CONCLUSION

This study evaluated the effects ethnic name partitioning has on AND using machine learning methods. For this, author name instances in four labeled datasets were disambiguated under two scenarios. First, similarity scores of instance pairs over four basic features—author name, coauthor names, paper title, and publication venue—were used to train and test disambiguation algorithms. Second, in addition to the basic features, ENGs were tagged to name instances to allow algorithms to build models that are optimized to each ENG. Comparisons of disambiguation performances before and after ENG‐aware disambiguation showed that using ethnic name partition can substantially improve algorithmic performances. Such performance improvements occurred across all ENGs, although performance gains and losses at each ENG level were observed in different ways depending on the types of measures—precision or recall—and target classifications—positive (match) or negative (nonmatch) pairs.

As detailed in the discussion above, ethnic name partition requires further research for us to better understand its impact on AND and apply it to disambiguation tasks for digital libraries that are struggling with authority control over fast‐growing ambiguous author names. This study is expected to motivate scholars and practitioners to study toward that direction by demonstrating the potential of ENG‐aware disambiguation in improving disambiguation performances.

## Supporting information

**Appendix** Supporting Information

## Endnotes

^1^
This new labeled dataset was created following the idea of a reviewer who suggested that the impact of ethnic name partitioning on AND may be confounded by the size differences of ambiguous names.

^2^

http://www.lbd.dcc.ufmg.br/lbd/collections/disambiguation/DBLP.tar.gz/at_download/file

^3^

http://arnetminer.org/lab-datasets/disambiguation/rich-author-disambiguation-data.zip

^4^

https://doi.org/10.7802/1234

^5^

https://www.nlm.nih.gov/bsd/medline.html

^6^

https://orcid.org/

^7^

https://github.com/stanfordnlp/CoreNLP/blob/master/data/edu/stanford/nlp/patterns/surface/stopwords.txt

^8^

https://tartarus.org/martin/PorterStemmer/

^9^

https://databank.illinois.edu/datasets/IDB-9087546

^10^
Twenty‐six ethnicities include African, Arab, Baltic, Caribbean, Chinese, Dutch, English, French, German, Greek, Hispanic, Hungarian, Indian, Indonesian, Israeli, Italian, Japanese, Korean, Mongolian, Nordic, Polynesian, Romanian, Slav, Thai, Turkish, and Vietnamese. In Ethnea, some name instances are assigned two ethnicities (e.g., “Jane Kim” → Korean‐English) if the surname and forename of an author name are associated frequently with different ethnicities.

^11^

https://doi.org/10.6084/m9.figshare.14043791

^12^
As only instances in the same block in which they share at least the same full name and first forename initial are compared, all the pairs in the block have the same ethnicity tag.
