# Supplementary material for: Ethnicity‐based name partitioning for author name disambiguation using supervised machine learning
Source: J Assoc Inf Sci Technol. 2021 Feb 23;72(8):979–94. doi: 10.1002/asi.24459 (PMC8359369; doi:10.1002/asi.24459)
Supplement: Supplementary file 1 — Appendix Supporting Information [file ASI-72-979-s001.docx]

Supplementary Material

This is a supplementary document for the paper entitled “Ethnicity-based name partitioning for author name disambiguation using supervised machine learning” by Jinseok Kim, Jenna Kim, and Jason Owen-Smith, which is published in the Journal of the Association for Information Science and Technology.

This document contains figures that report (1) disambiguation performances per ethnic name group and (2) feature similarity score distributions in four labeled data.

**List of Contents**

p.3) Figure S1: Disambiguation Performances per ENG ‘Before’ Versus ‘After’ ENG-Aware Disambiguation by Logistic Regression on AMINER

p.4) Figure S2: Disambiguation Performances per ENG ‘Before’ Versus ‘After’ ENG-Aware Disambiguation by Naïve Bayes on AMINER

p.5) Figure S3: Disambiguation Performances per ENG ‘Before’ Versus ‘After’ ENG-Aware Disambiguation by Random Forest on AMINER

p.6) Figure S4: Disambiguation Performances per ENG ‘Before’ Versus ‘After’ ENG-Aware Disambiguation by Gradient Boosting on AMINER

p.7) Figure S5: Disambiguation Performances per ENG ‘Before’ Versus ‘After’ ENG-Aware Disambiguation by Logistic Regression on GESIS

p.8) Figure S6: Disambiguation Performances per ENG ‘Before’ Versus ‘After’ ENG-Aware Disambiguation by Naïve Bayes on GESIS

p.9) Figure S7: Disambiguation Performances per ENG ‘Before’ Versus ‘After’ ENG-Aware Disambiguation by Random Forest on GESIS

p.10) Figure S8: Disambiguation Performances per ENG ‘Before’ Versus ‘After’ ENG-Aware Disambiguation by Gradient Boosting on GESIS

p.11) Figure S9: Disambiguation Performances per ENG ‘Before’ Versus ‘After’ ENG-Aware Disambiguation by Logistic Regression on UM-IRIS

p.12) Figure S10: Disambiguation Performances per ENG ‘Before’ Versus ‘After’ ENG-Aware Disambiguation by Naïve Bayes on UM-IRIS

p.13) Figure S11: Disambiguation Performances per ENG ‘Before’ Versus ‘After’ ENG-Aware Disambiguation by Random Forest on UM-IRIS

p.14) Figure S12: Disambiguation Performances per ENG ‘Before’ Versus ‘After’ ENG-Aware Disambiguation by Gradient Boosting on UM-IRIS

p.15) Figure S13: Disambiguation Performances per ENG ‘Before’ Versus ‘After’ ENG-Aware Disambiguation by Logistic Regression on KISTI

p.16) Figure S14: Disambiguation Performances per ENG ‘Before’ Versus ‘After’ ENG-Aware Disambiguation by Naïve Bayes on KISTI

p.17) Figure S15: Disambiguation Performances per ENG ‘Before’ Versus ‘After’ ENG-Aware Disambiguation by Random Forest on KISTI

p.18) Figure S16: Disambiguation Performances per ENG ‘Before’ Versus ‘After’ ENG-Aware Disambiguation by Gradient Boosting on KISTI

p.19) Figure S17: Feature Similarity Score Distributions per ENG for Positive and Negative Pairs in AMINER Test Data

p.20) Figure S18: Feature Similarity Score Distributions per ENG for Positive and Negative Pairs in GESIS Test Data

p.21) Figure S19: Feature Similarity Score Distributions per ENG for Positive and Negative Pairs in UM-IRIS Test Data

p.22) Figure S20: Feature Similarity Score Distributions per ENG for Positive and Negative Pairs in KISTI Test Data


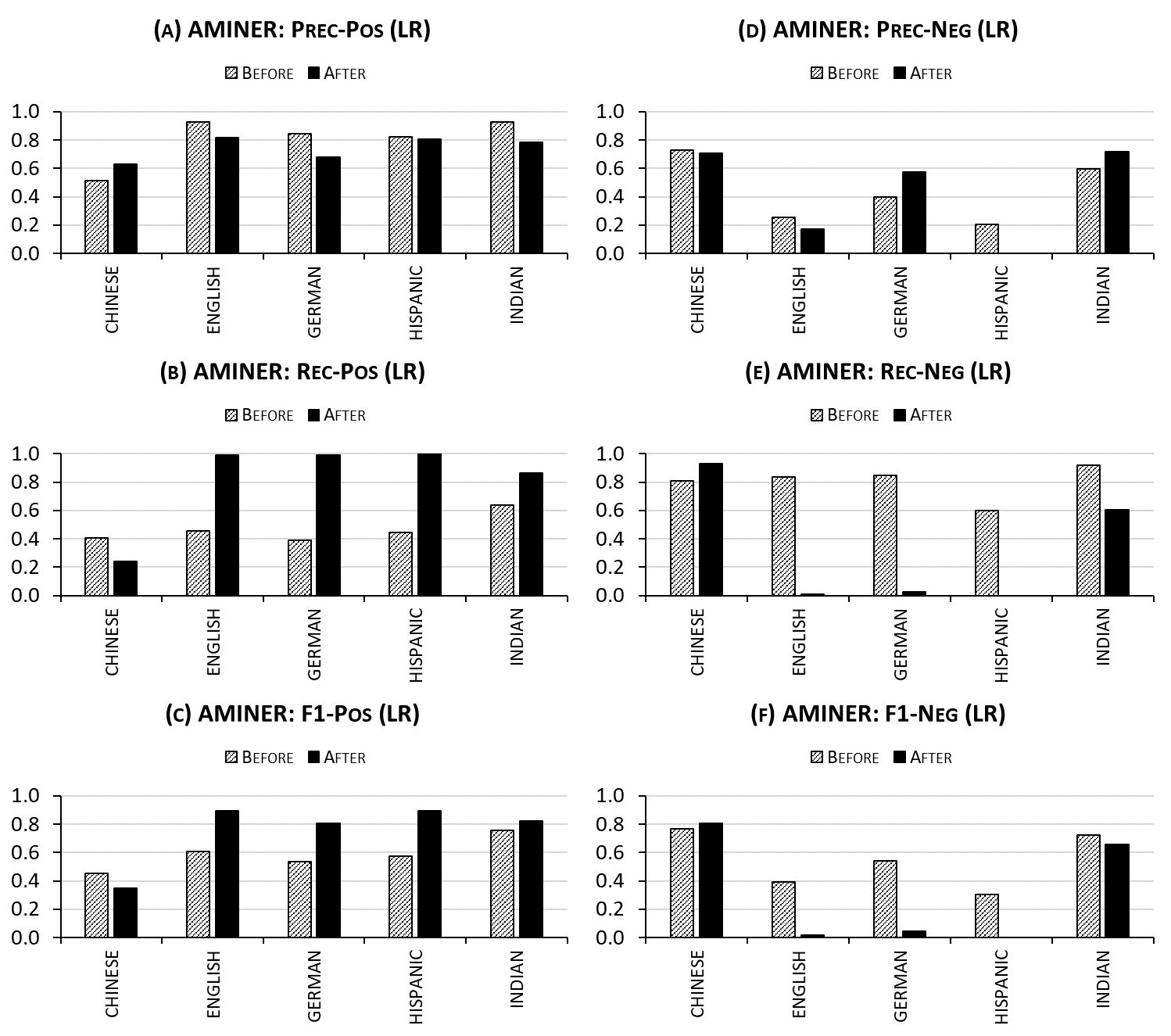


Figure S1: Disambiguation Performances per ENG ‘Before’ Versus ‘After’ ENG-Aware Disambiguation by Logistic Regression on AMINER


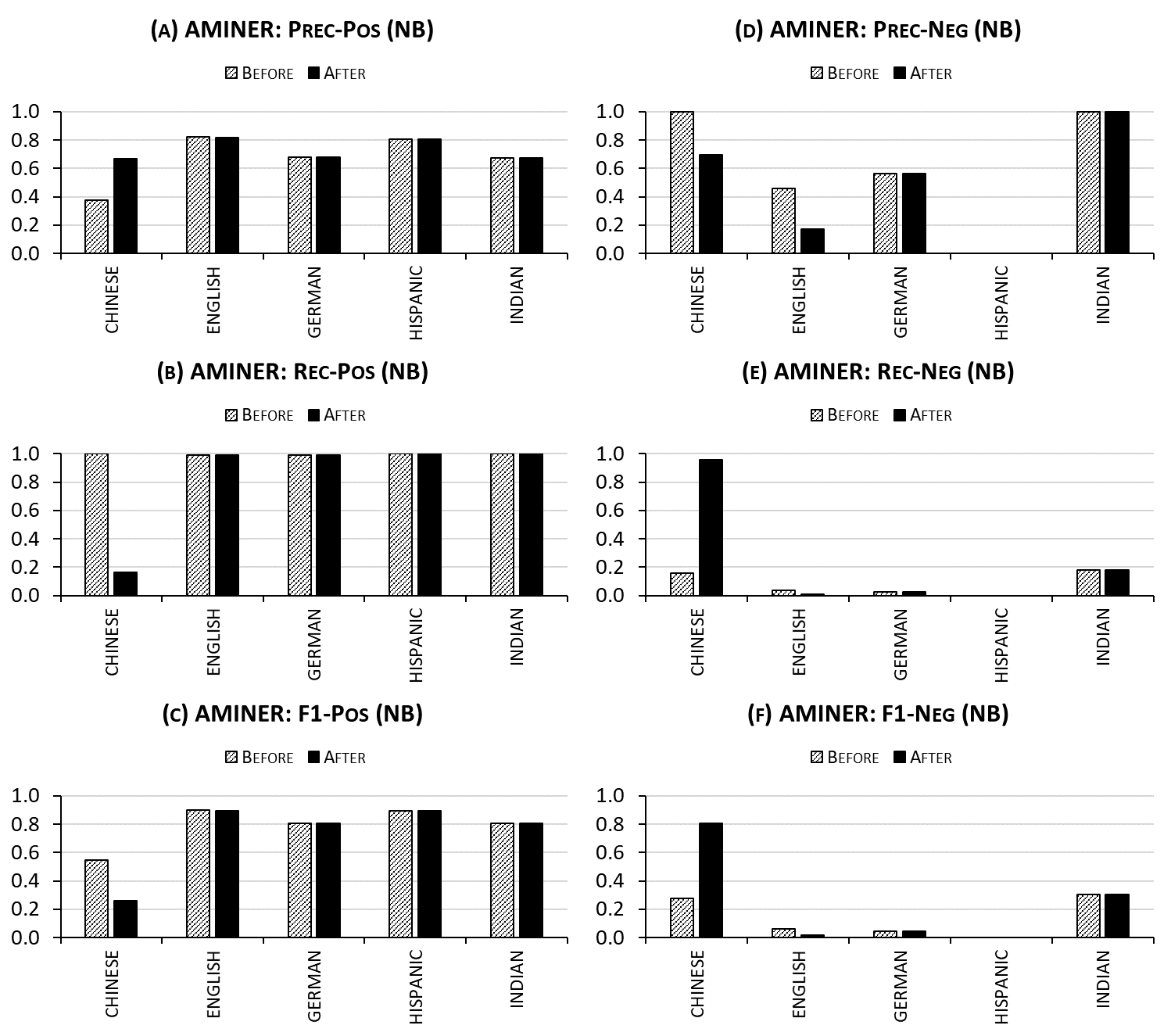


Figure S2: Disambiguation Performances per ENG ‘Before’ Versus ‘After’ ENG-Aware Disambiguation by Naïve Bayes on AMINER


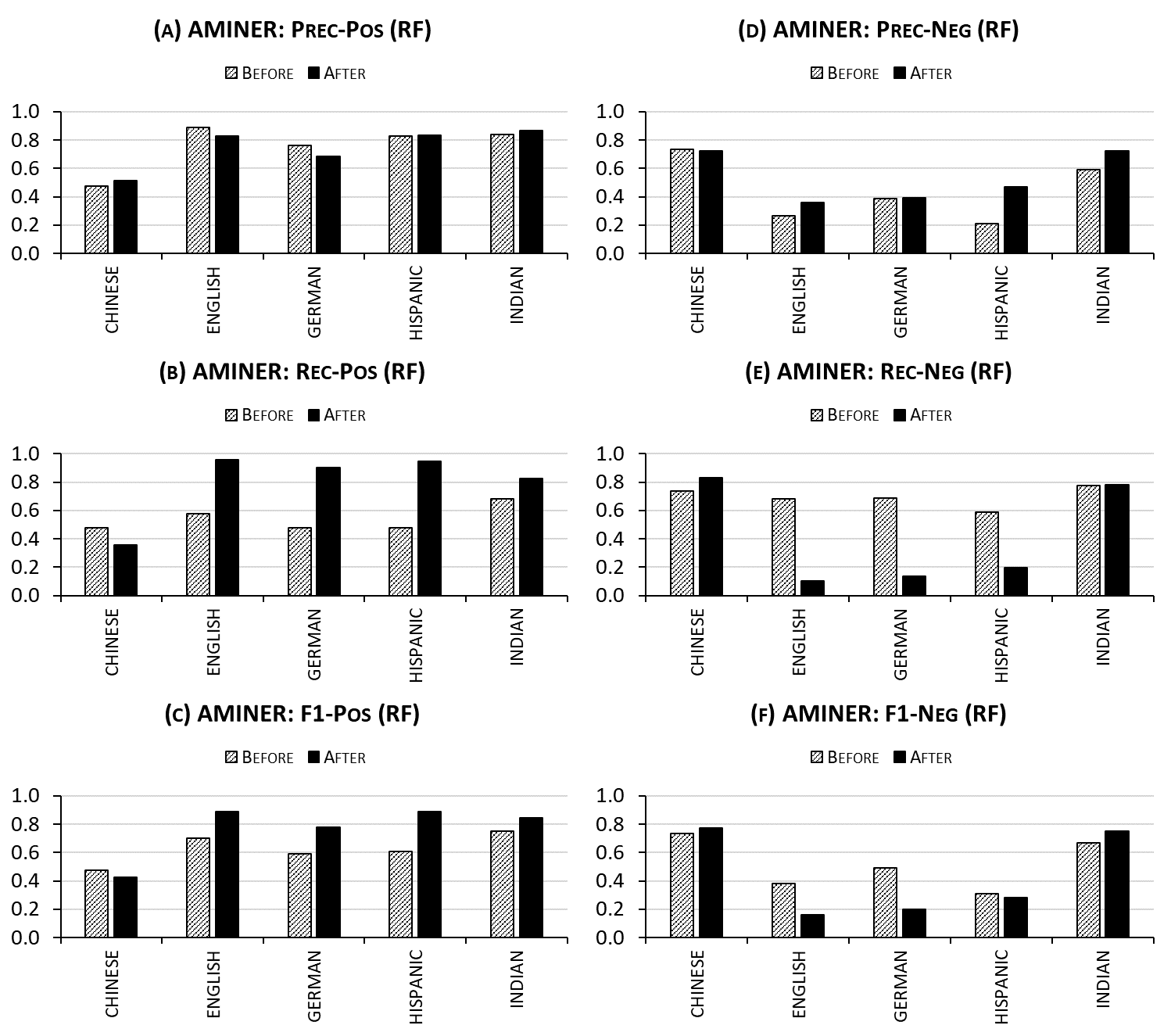


Figure S3: Disambiguation Performances per ENG ‘Before’ Versus ‘After’ ENG-Aware Disambiguation by Random Forest on AMINER


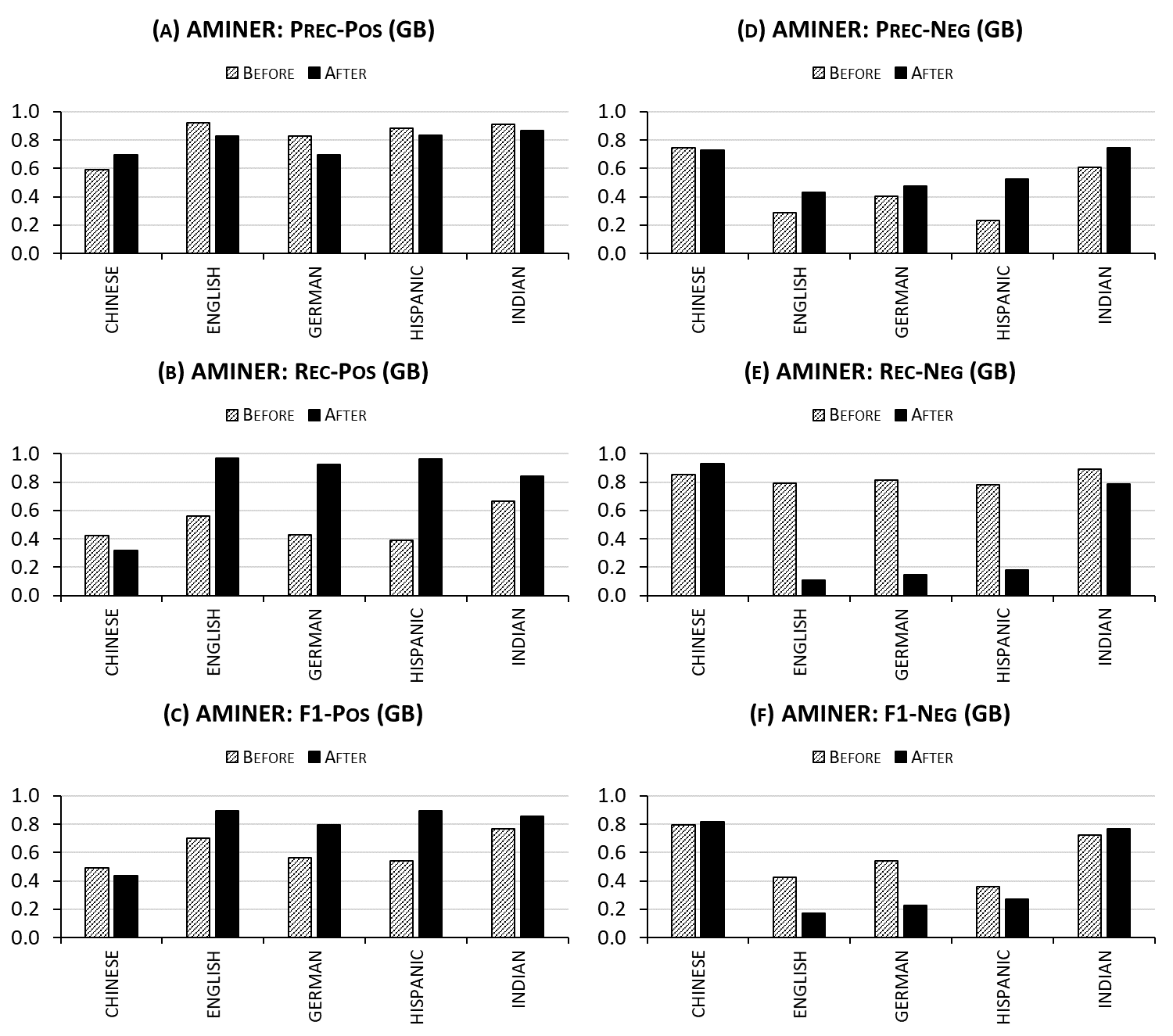


Figure S4: Disambiguation Performances per ENG ‘Before’ Versus ‘After’ ENG-Aware Disambiguation by Gradient Boosting on AMINER


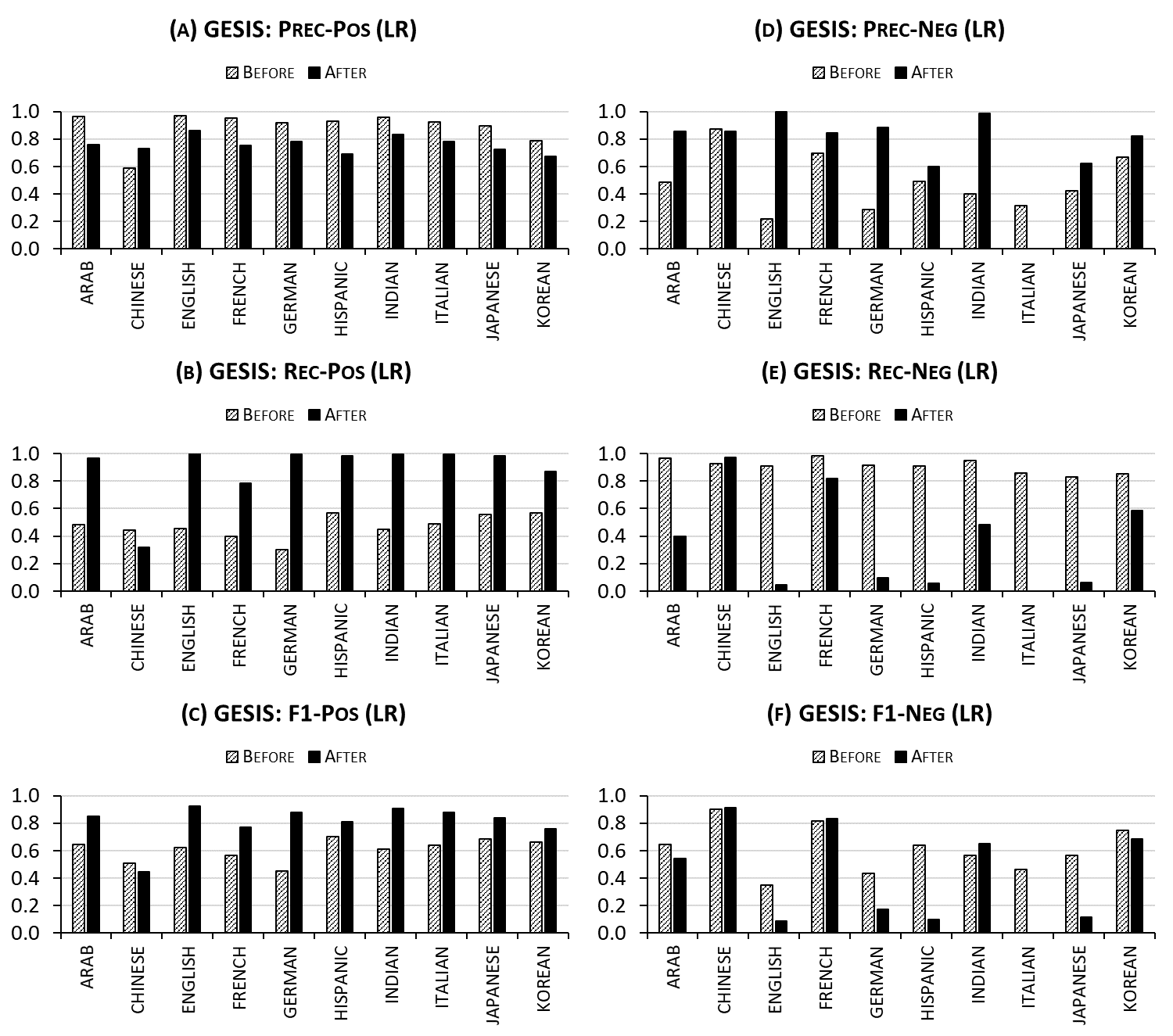


Figure S5: Disambiguation Performances per ENG ‘Before’ Versus ‘After’ ENG-Aware Disambiguation by Logistic Regression on GESIS


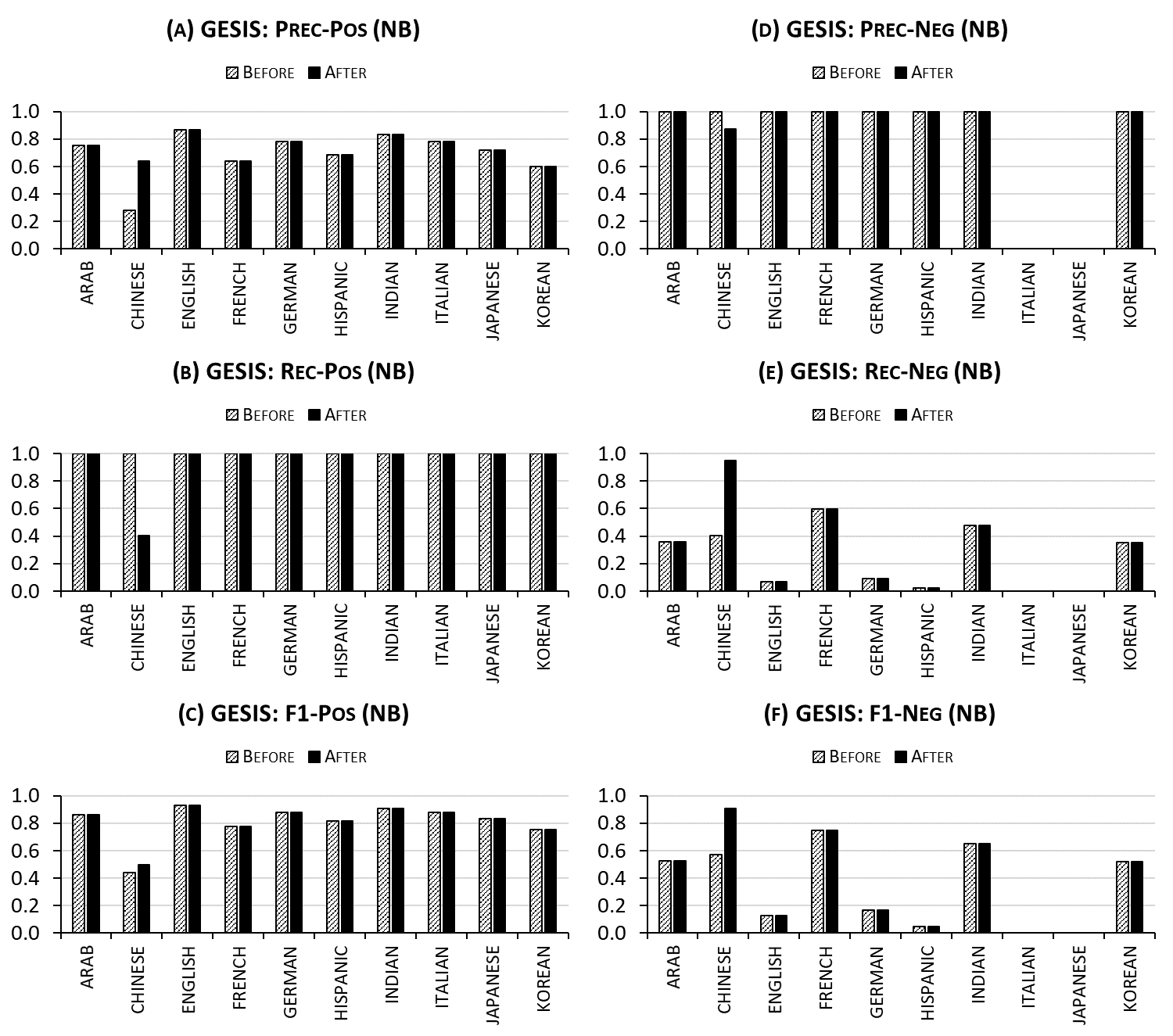


Figure S6: Disambiguation Performances per ENG ‘Before’ Versus ‘After’ ENG-Aware Disambiguation by Naïve Bayes on GESIS


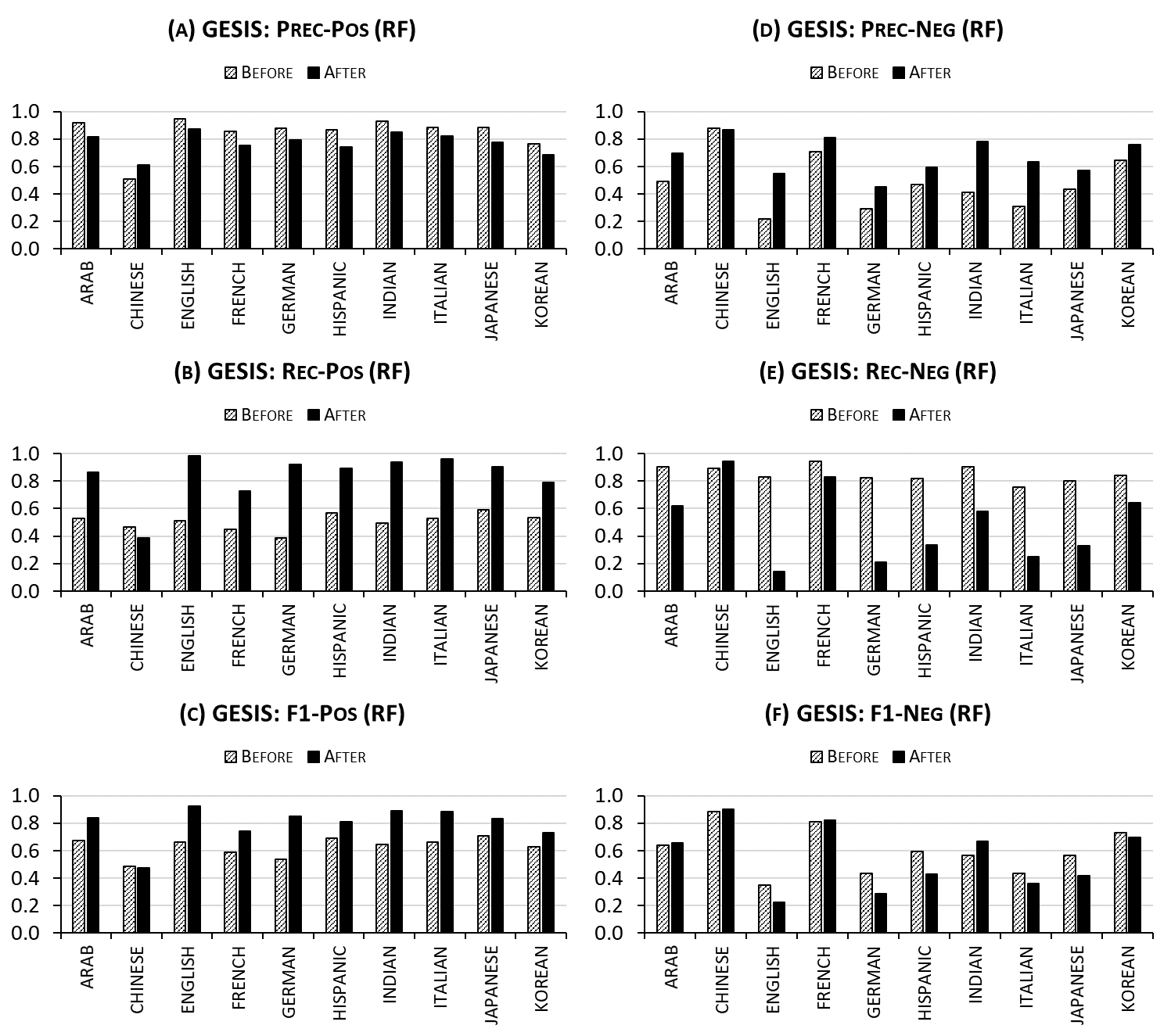


Figure S7: Disambiguation Performances per ENG ‘Before’ Versus ‘After’ ENG-Aware Disambiguation by Random Forest on GESIS


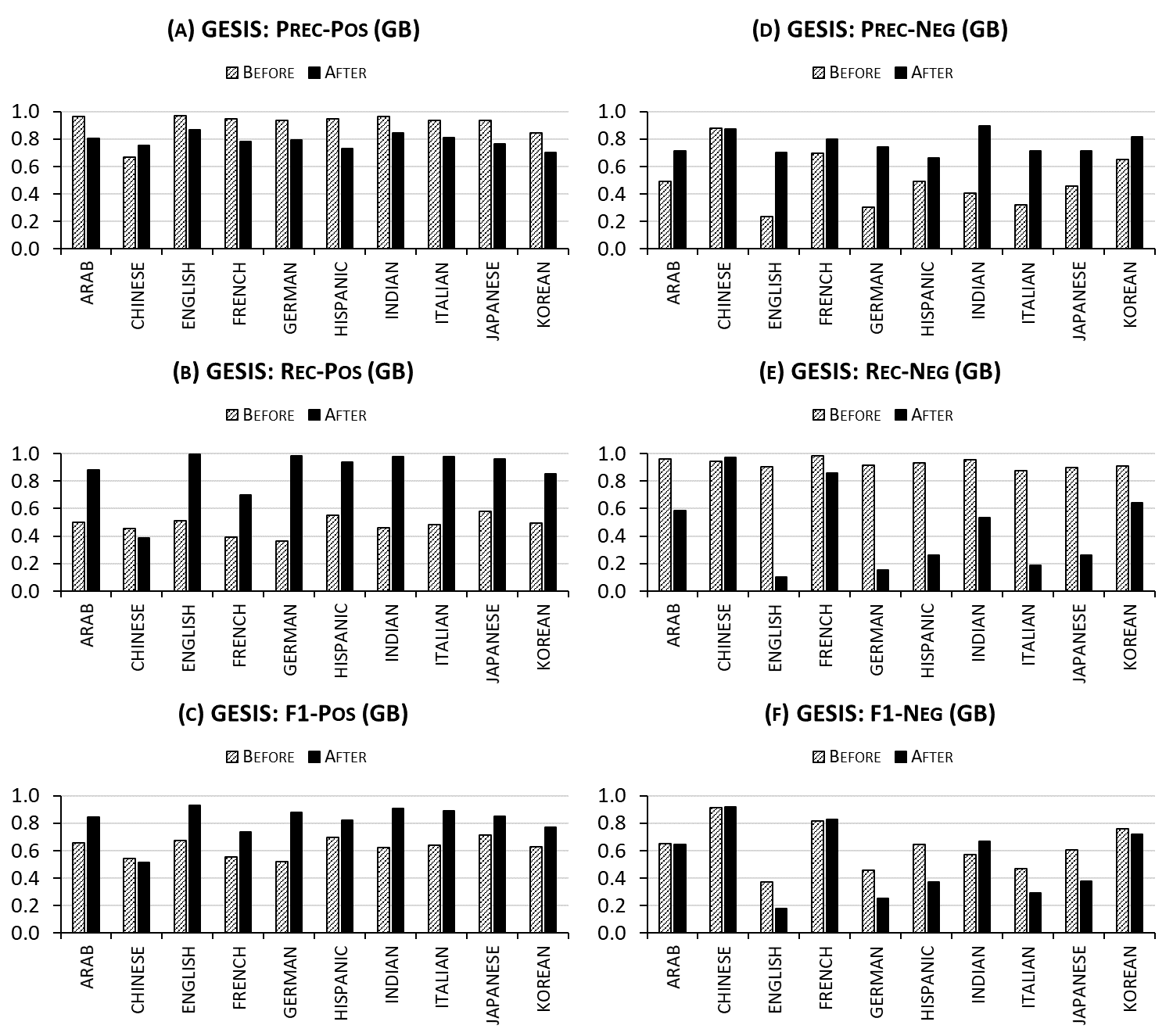


Figure S8: Disambiguation Performances per ENG ‘Before’ Versus ‘After’ ENG-Aware Disambiguation by Gradient Boosting on GESIS


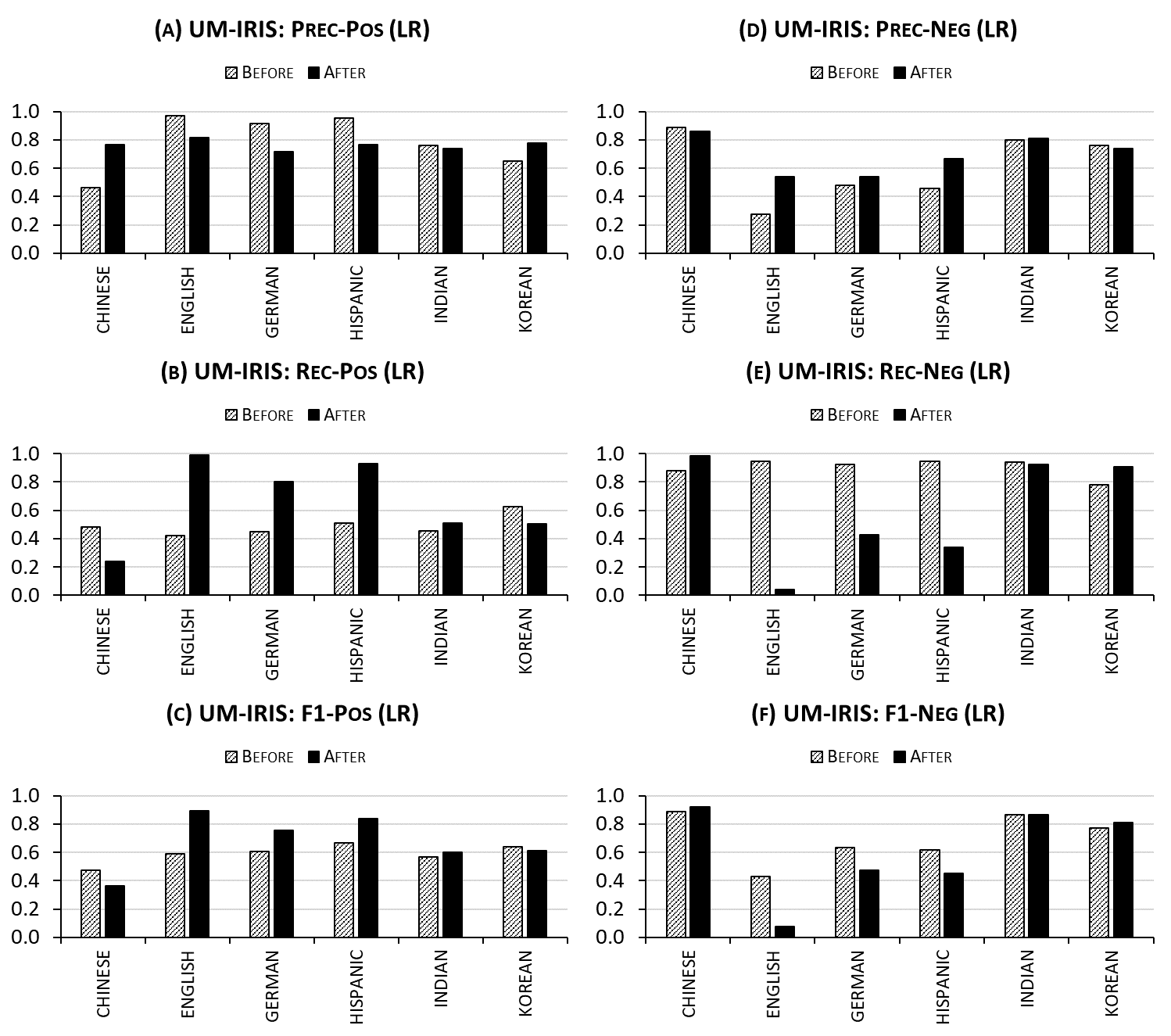


Figure S9: Disambiguation Performances per ENG ‘Before’ Versus ‘After’ ENG-Aware Disambiguation by Logistic Regression on UM-IRIS


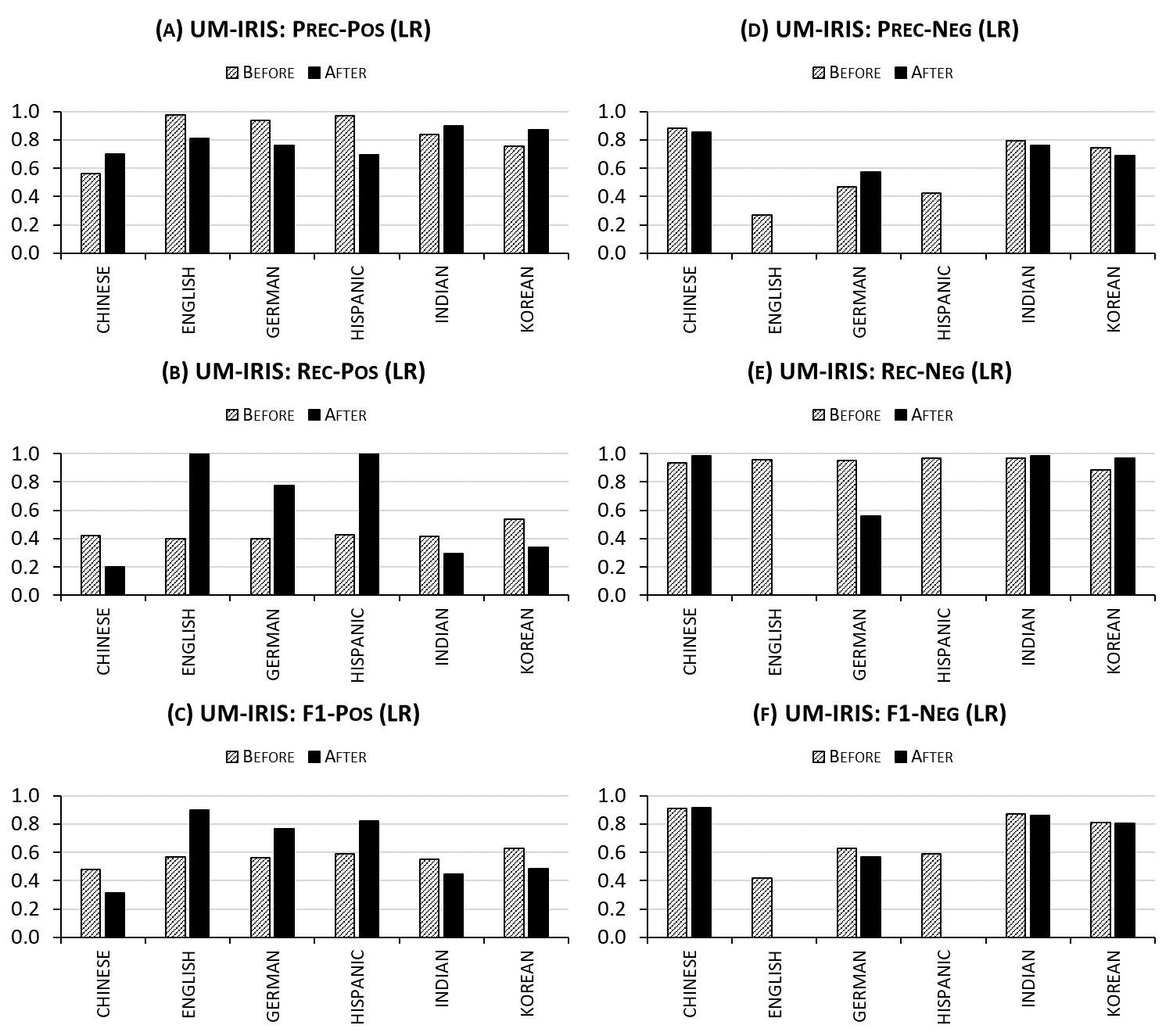


Figure S10: Disambiguation Performances per ENG ‘Before’ Versus ‘After’ ENG-Aware Disambiguation by Naïve Bayes on UM-IRIS


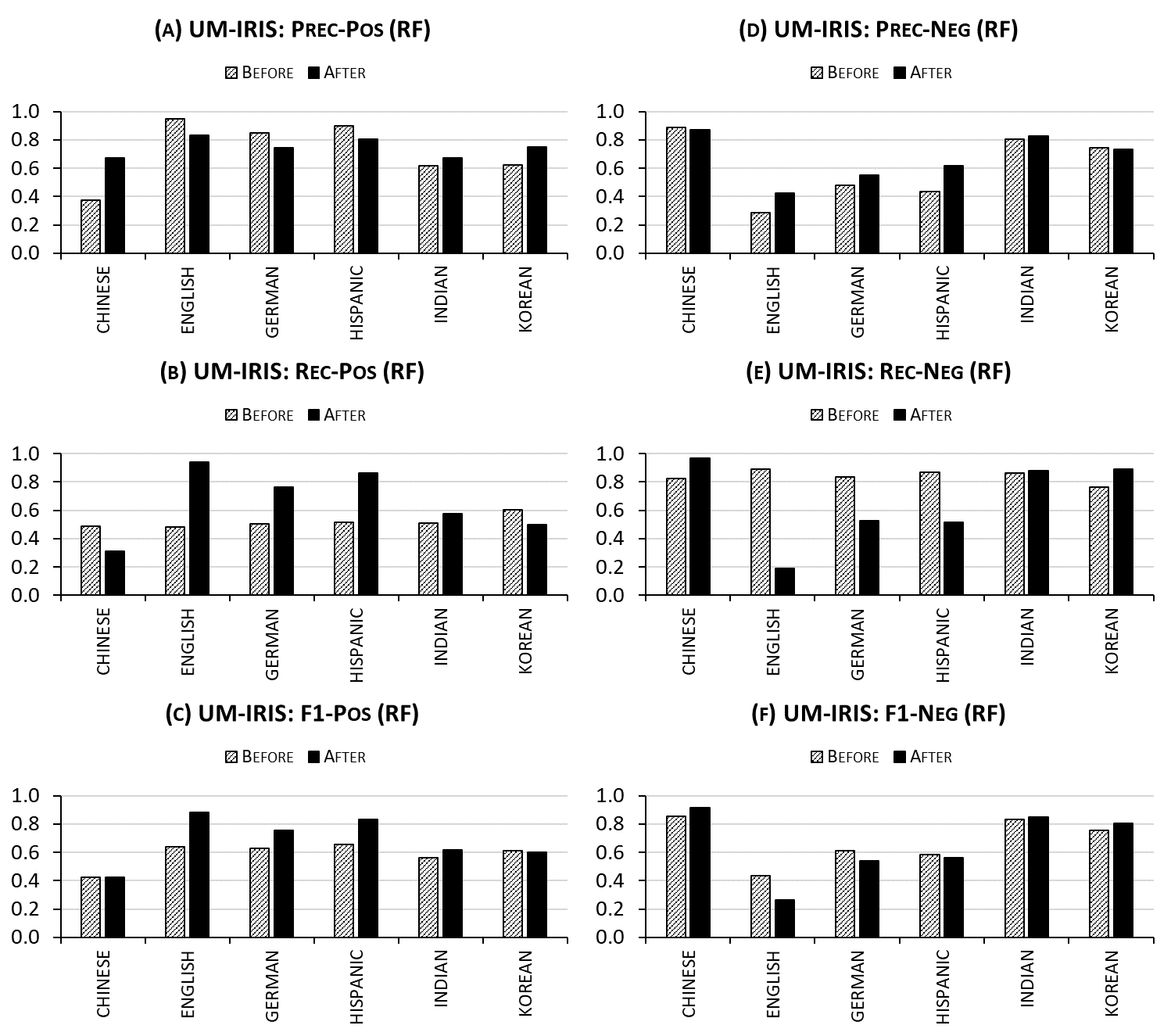


Figure S11: Disambiguation Performances per ENG ‘Before’ Versus ‘After’ ENG-Aware Disambiguation by Random Forest on UM-IRIS


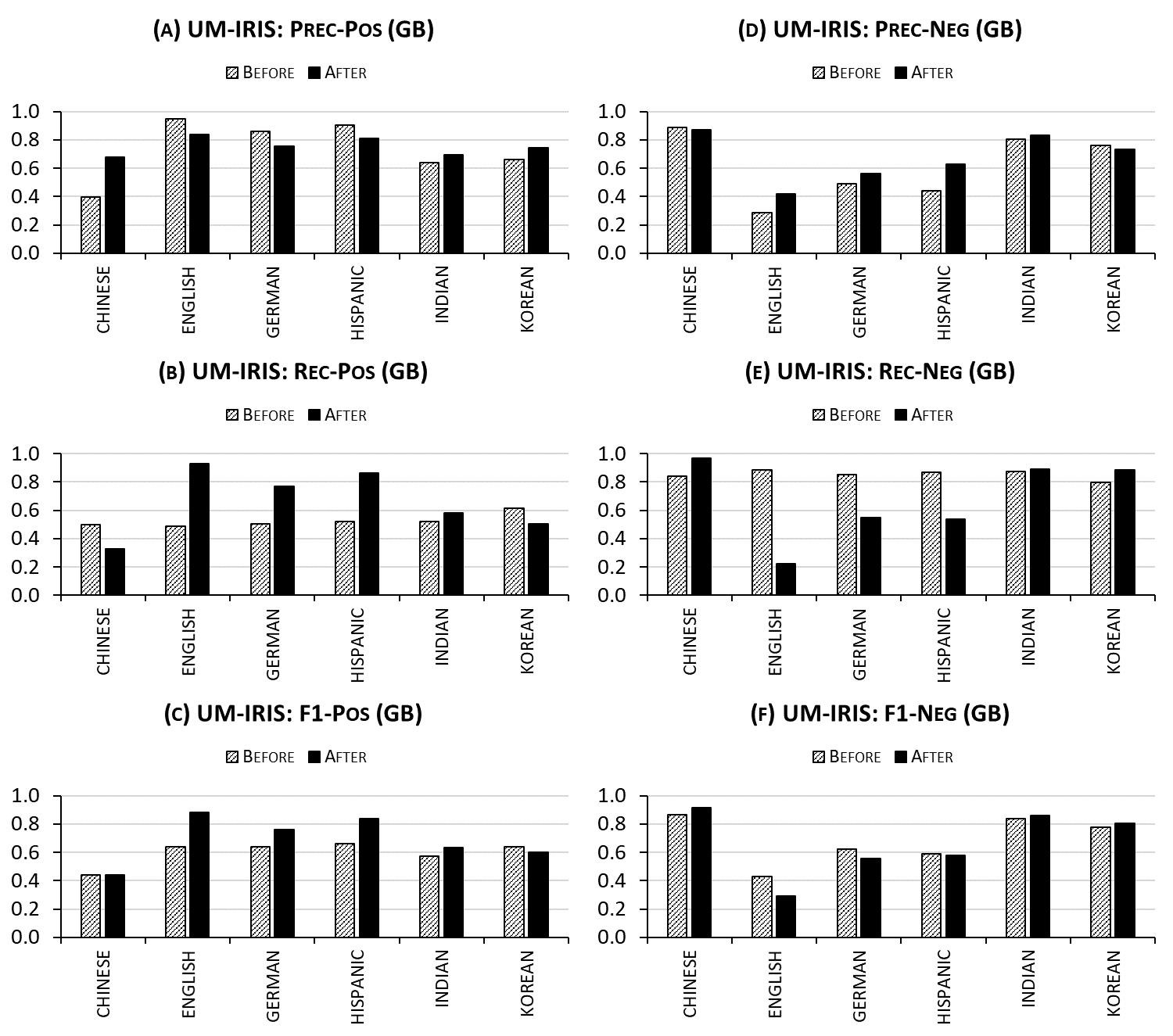


Figure S12: Disambiguation Performances per ENG ‘Before’ Versus ‘After’ ENG-Aware Disambiguation by Gradient Boosting on UM-IRIS


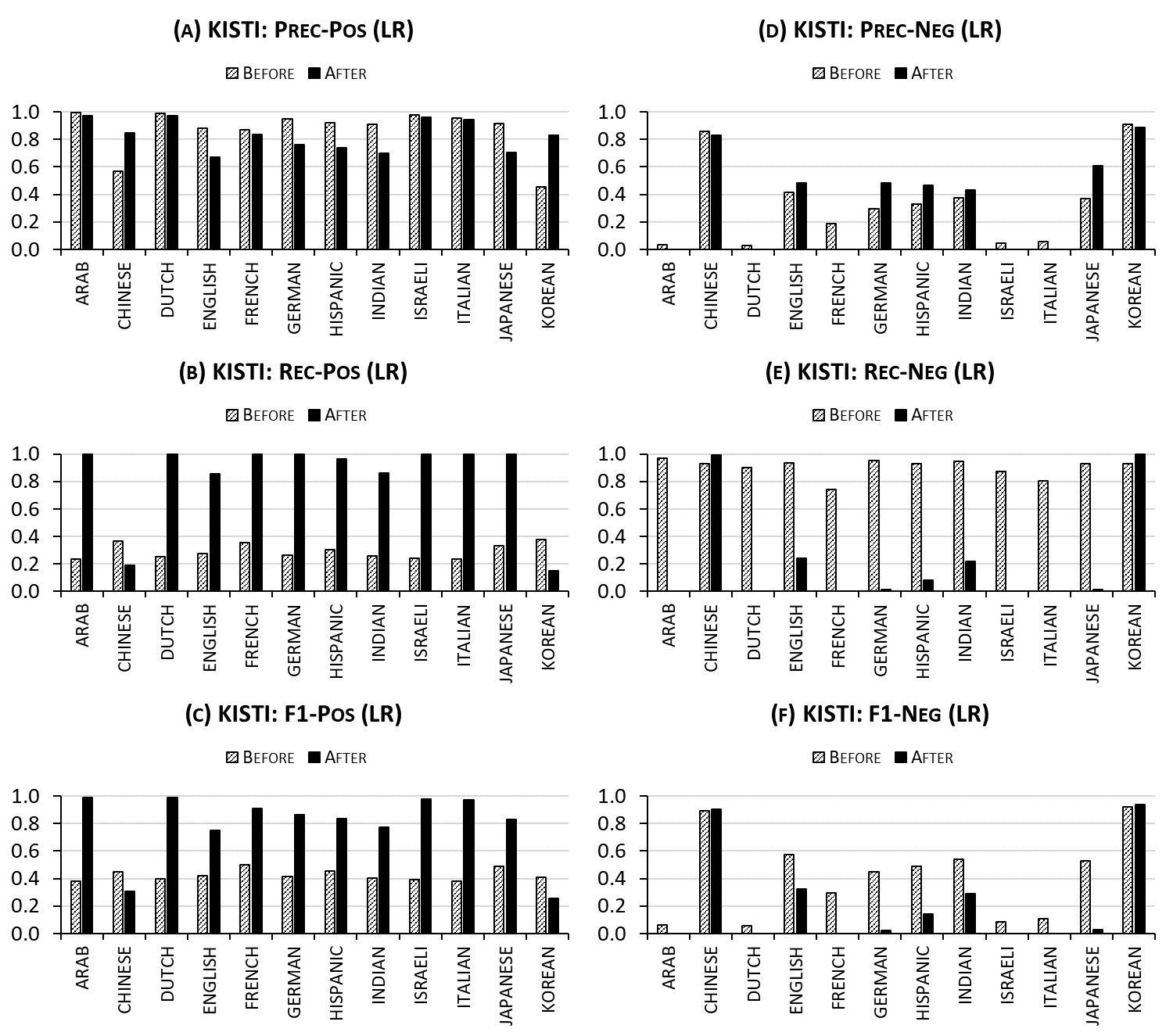


Figure S13: Disambiguation Performances per ENG ‘Before’ Versus ‘After’ ENG-Aware Disambiguation by Logistic Regression on KISTI


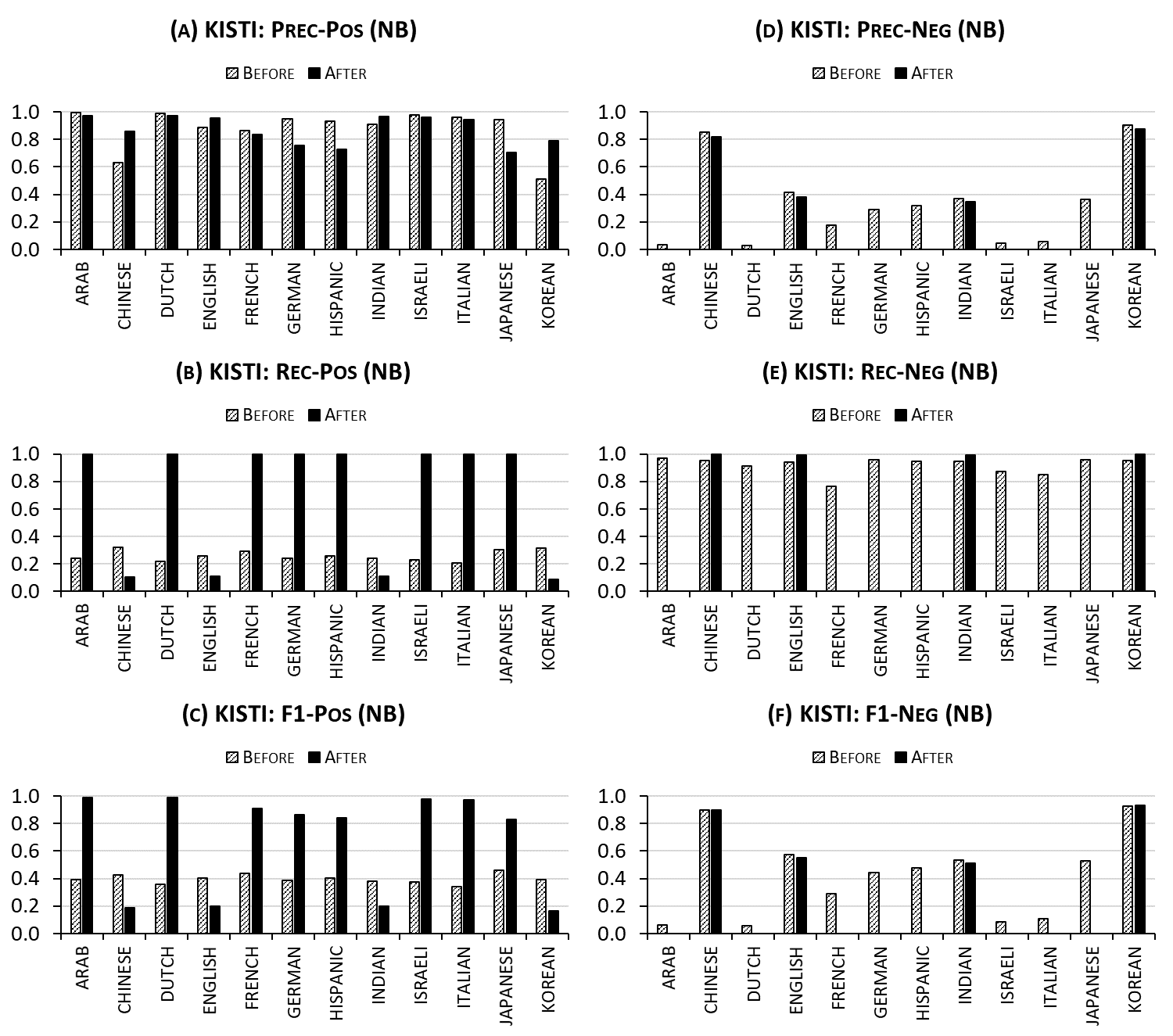


Figure S14: Disambiguation Performances per ENG ‘Before’ Versus ‘After’ ENG-Aware Disambiguation by Naïve Bayes on KISTI


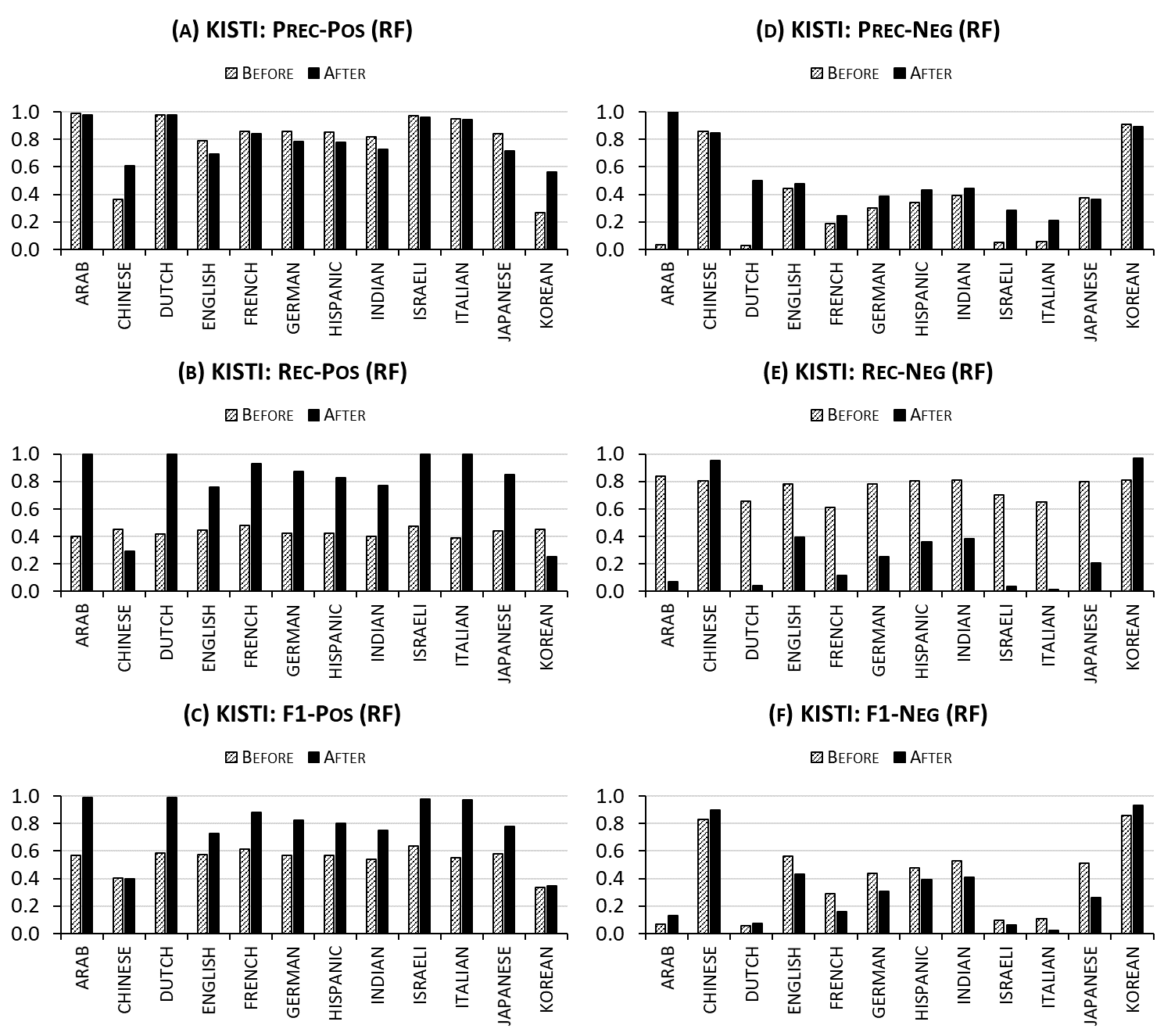


Figure S15: Disambiguation Performances per ENG ‘Before’ Versus ‘After’ ENG-Aware Disambiguation by Random Forest on KISTI


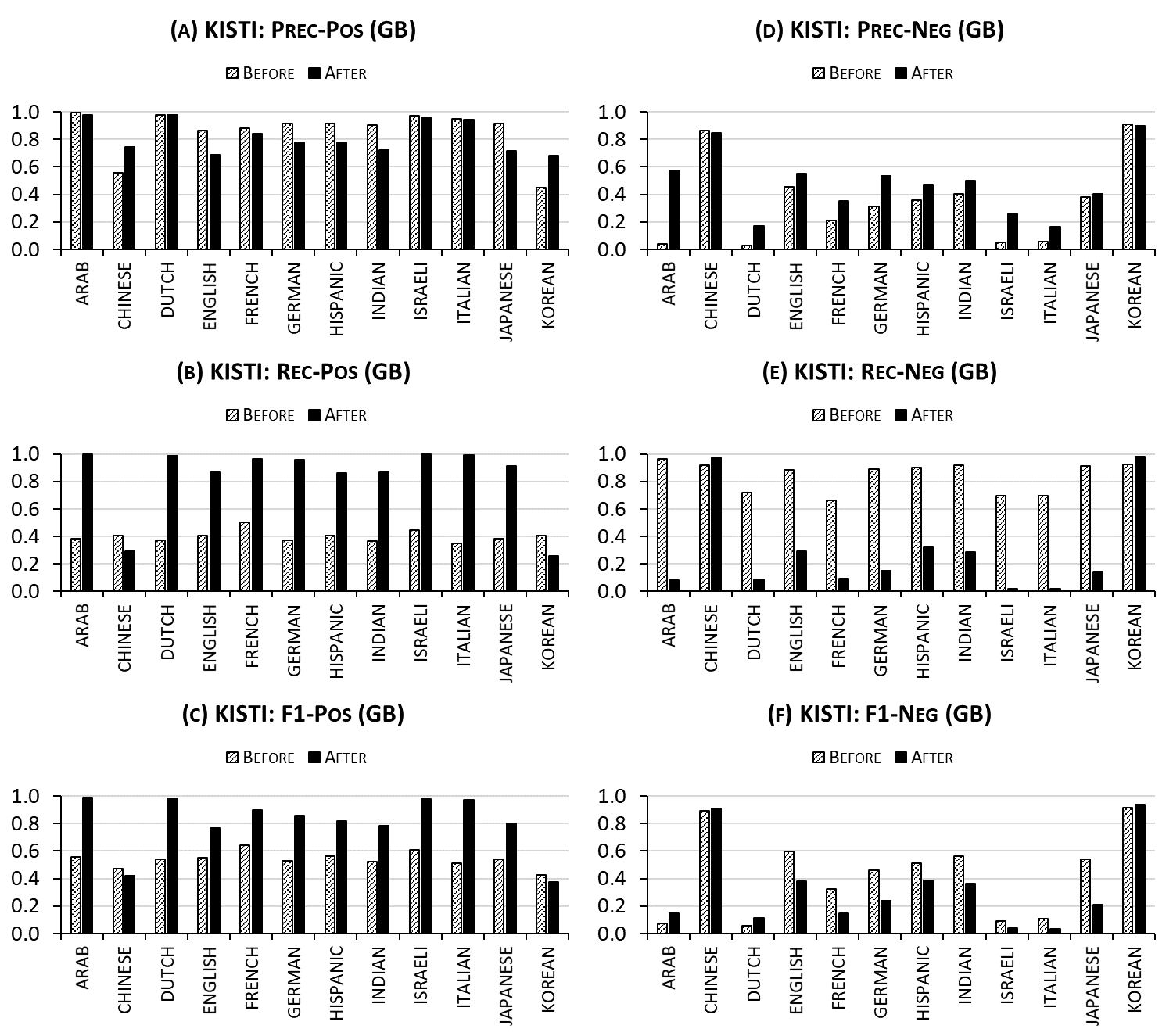


Figure S16: Disambiguation Performances per ENG ‘Before’ Versus ‘After’ ENG-Aware Disambiguation by Gradient Boosting on KISTI


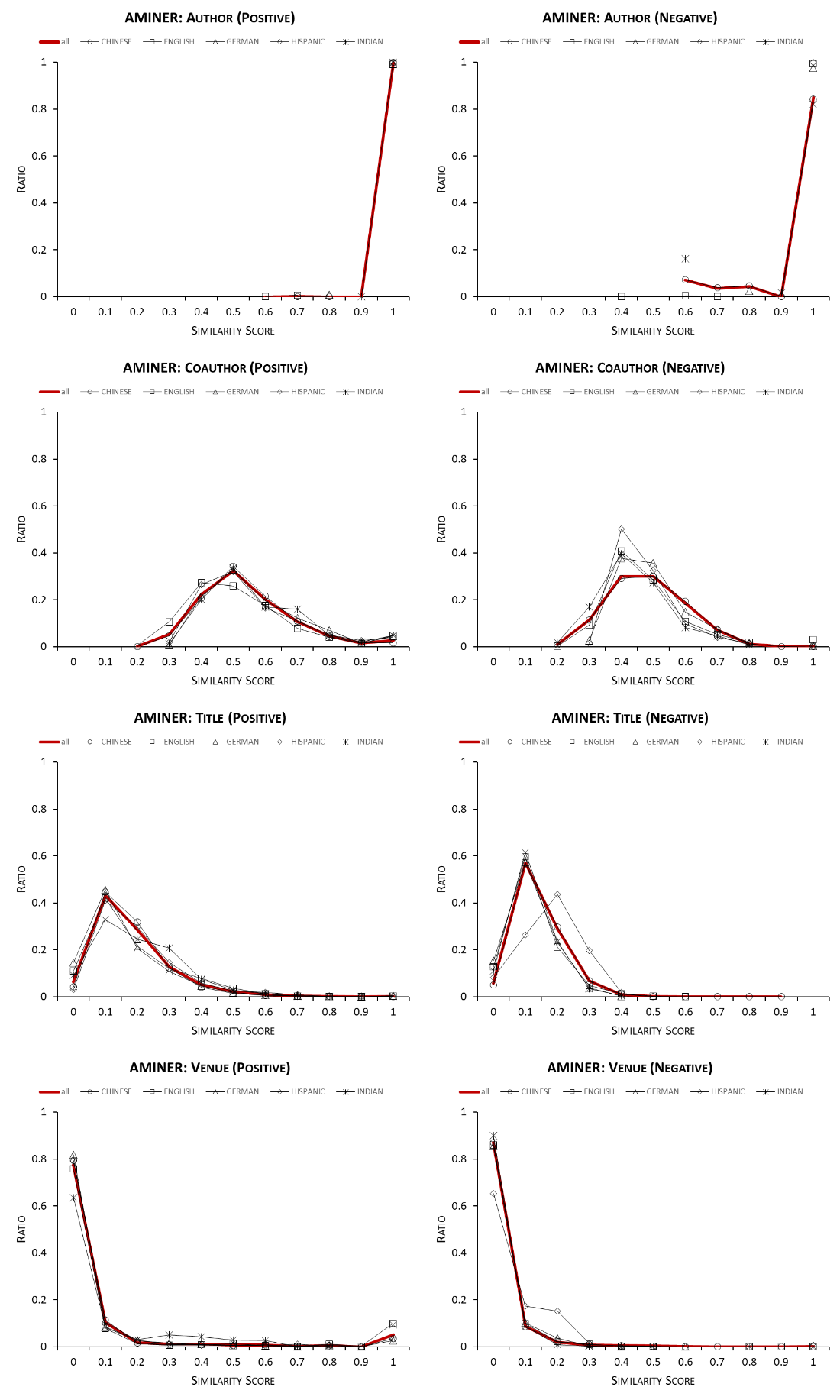


Figure S17: Feature Similarity Score Distributions per ENG for Positive and Negative Pairs in AMINER Test Data


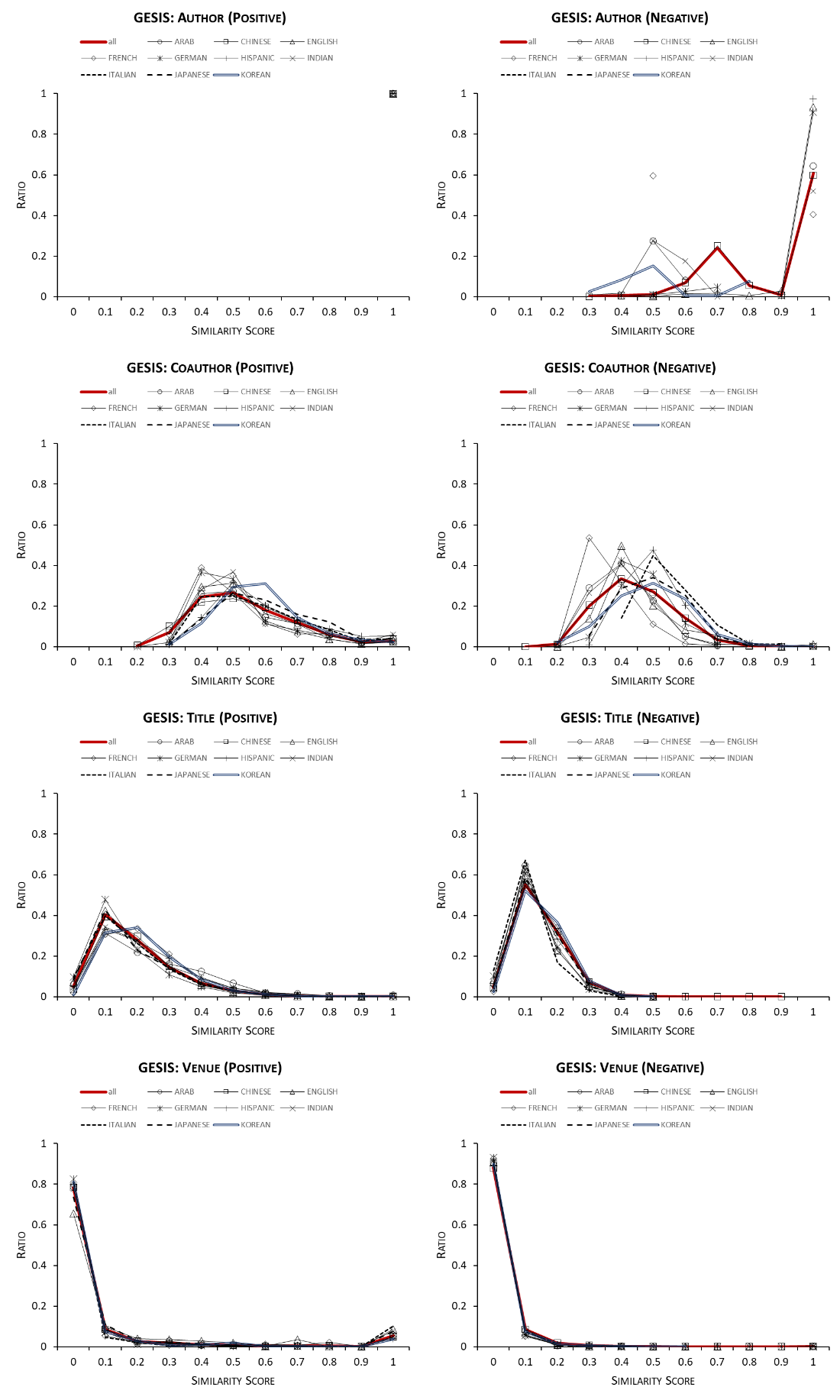


Figure S18: Feature Similarity Score Distributions per ENG for Positive and Negative Pairs in GESIS Test Data


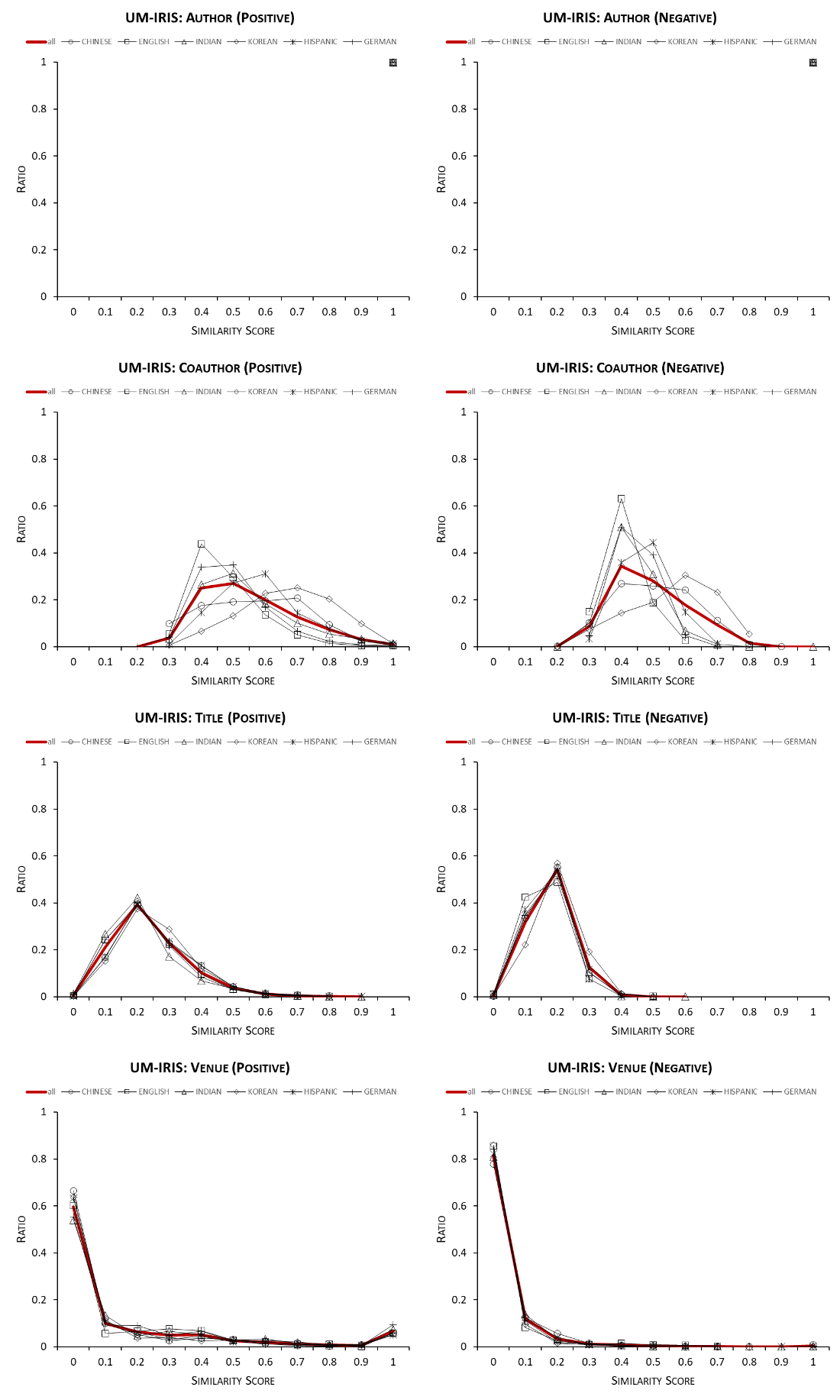


Figure S19: Feature Similarity Score Distributions per ENG for Positive and Negative Pairs in UM-IRIS Test Data


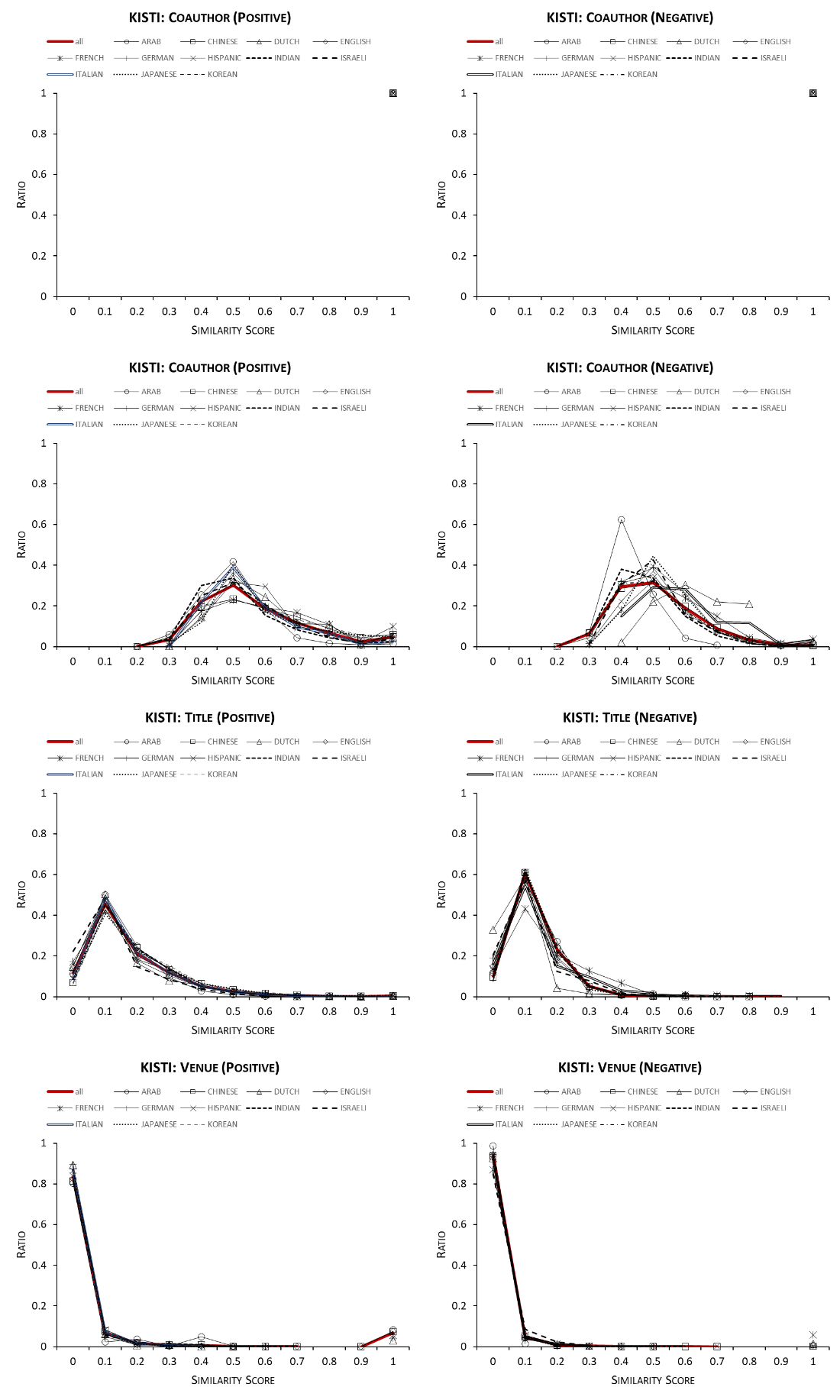


Figure S20: Feature Similarity Score Distributions per ENG for Positive and Negative Pairs in KISTI Test Data
